# The pelagic food web of the Western Adriatic Sea: a focus on the role of small pelagics

**DOI:** 10.1038/s41598-023-40665-w

**Published:** 2023-09-04

**Authors:** E. Fanelli, Z. Da Ros, S. Menicucci, S. Malavolti, I. Biagiotti, G. Canduci, A. De Felice, I. Leonori

**Affiliations:** 1 Department of Life and Environmental Sciences, Polytechnic University of Marche, Via Brecce Bianche, 60131 Ancona, Italy; 2 Stazione Zoologica Anton Dohrn, Villa Comunale, Naples, Italy; 3 Institute for Marine Biological Resources and Biotechnologies (IRBIM), National Research Council (CNR), Largo Fiera Della Pesca, 60125 Ancona, Italy; 4 Alma Mater Studiorum, Università di Bologna, Via Zamboni, 33, 40126 Bologna, Italy

**Keywords:** Ecology, Ecology, Ocean sciences

## Abstract

The Adriatic Sea is one of the largest areas of occurrence of shared small pelagic stocks and the most fished area of the Mediterranean Sea, which is in turn one of the most exploited basins of the world. The variations in the stable isotope contents (*δ*^15^N and *δ*^13^C) were determined for three small pelagic fishes (i.e., *Engraulis encrasicolus*, *Sardina pilchardus*, and *Sprattus sprattus*, respectively known as anchovies, sardines and sprats) collected across the western side of the basin. Our data allowed to determine the width and features of their trophic niches, to assess potential overlap or resource partitioning among them, and likely anticipate species adaptation to future climate change scenarios. Moreover, variations in stable isotope contents were correlated to both resource availability (i.e., mesozooplankton) and environmental variables. The high productivity and in turn the high resource availability of the basin, especially in the northern part, resulted in favor of the resource partitioning that occurs in each sub-area of the Adriatic Sea among the three species. Medium-sized specimens of the three species mostly fed on small zooplankton, while adult sprats relied on large copepods and those of sardines and anchovies also consumed large portion of phytoplankton, confirming the high trophic plasticity of these two dominants small pelagic species. However, considering that anchovies have the greatest degree of trophic diversity compared with the other two species, they could be the most adapted to changing feeding conditions. The increase in sea temperatures that are reducing primary production and in turn zooplankton abundances, coupled with even more frequent extreme meteorologic events could exacerbate the competition for trophic resources among pelagic mesopredators, and could lead to more notable stocks’ fluctuations and unpredictable wasp-waist effects.

## Introduction

Small pelagic fishes represent most of the fish biomass in pelagic ecosystems but, despite this, their trophic level is usually dominated by only few species^1^. In the Mediterranean Sea, small pelagics are mostly represented by the European anchovy (*Engraulis encrasicolus*, Linnaeus, 1758), European sardine (*Sardina pilchardus,* Walbaum, 1792), European sprat (*Sprattus sprattus*, Linnaeus, 1758) and round sardinella (*Sardinella aurita*, Valenciennes, 1847).

**Figure 1:**
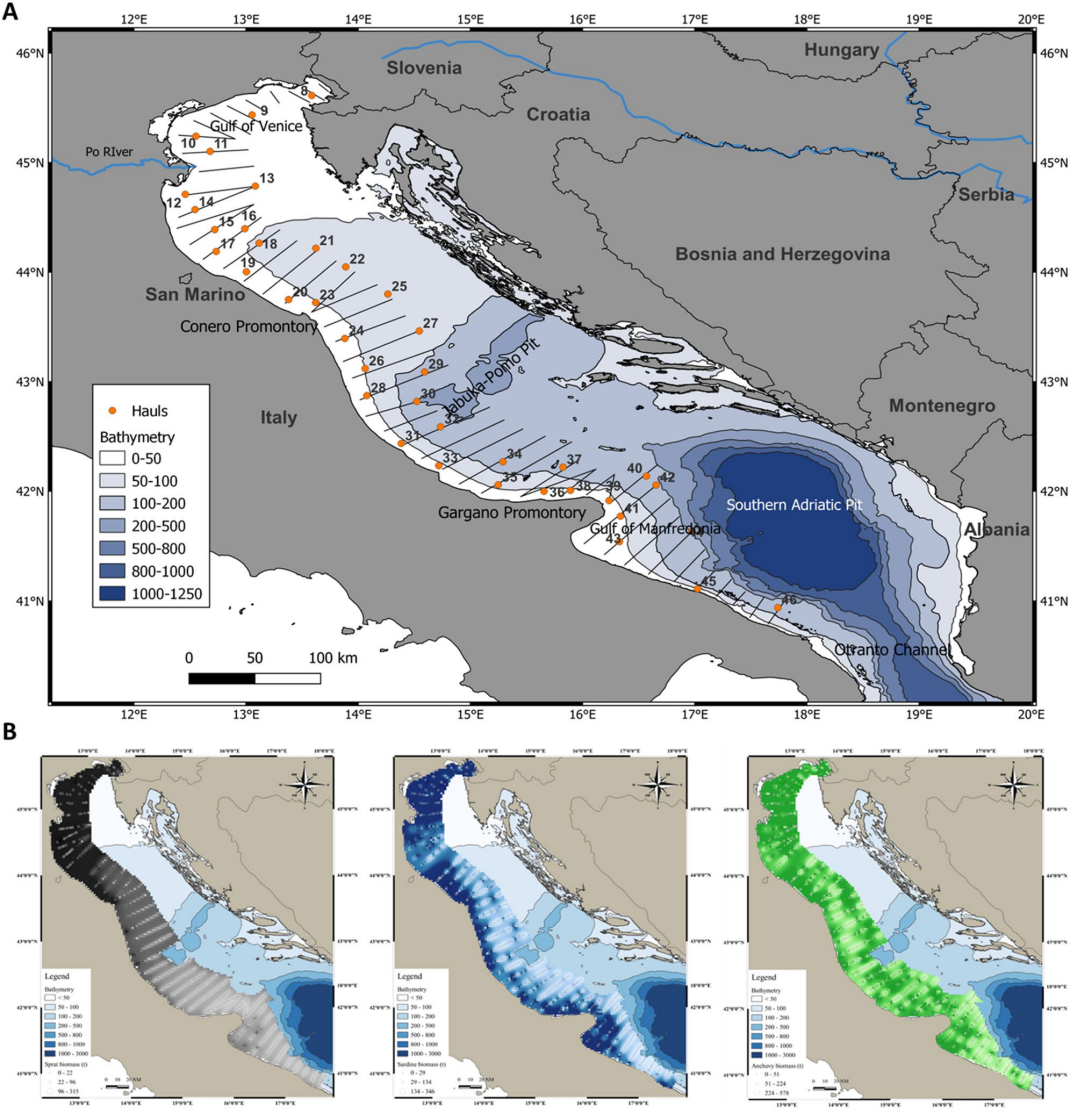
(**A**) Map of the sampling sites surveyed in 2019 for GSA 17 and GSA 18. Black lines represent transects for acoustic data sampling; orange dots are the hauls where small pelagic fish were collected. (**B**) Distribution maps of fish biomass (in tons, t) as derived by acoustic data from MEDIAS 2019 survey for *Sprattus sprattus* (on the left, black symbols), *Sardina pilchardus* (in the center, blue symbols), and *Engraulis encrasicolus* (on the right, green symbols). This figure was originally created with QGis version 3.28.8 (QGIS Development Team, 2023. QGIS Geographic Information System, Open Source Geospatial Foundation Project, http://qgis.osgeo.org.

This study is focused on sprats, anchovies, and sardines. Anchovy and sardine are widely distributed in the Eastern Atlantic Ocean and are common in the Mediterranean and Black Sea^2^, while sprat is mostly confined to the northeast Atlantic, and in the Mediterranean is concentrated in the Gulf of Lions, the Northern Adriatic, and the Black Sea^2^. In the Western Adriatic Sea, where this study was conducted, they share the same distribution area (Fig. 1A,B).

**Figure 2:**
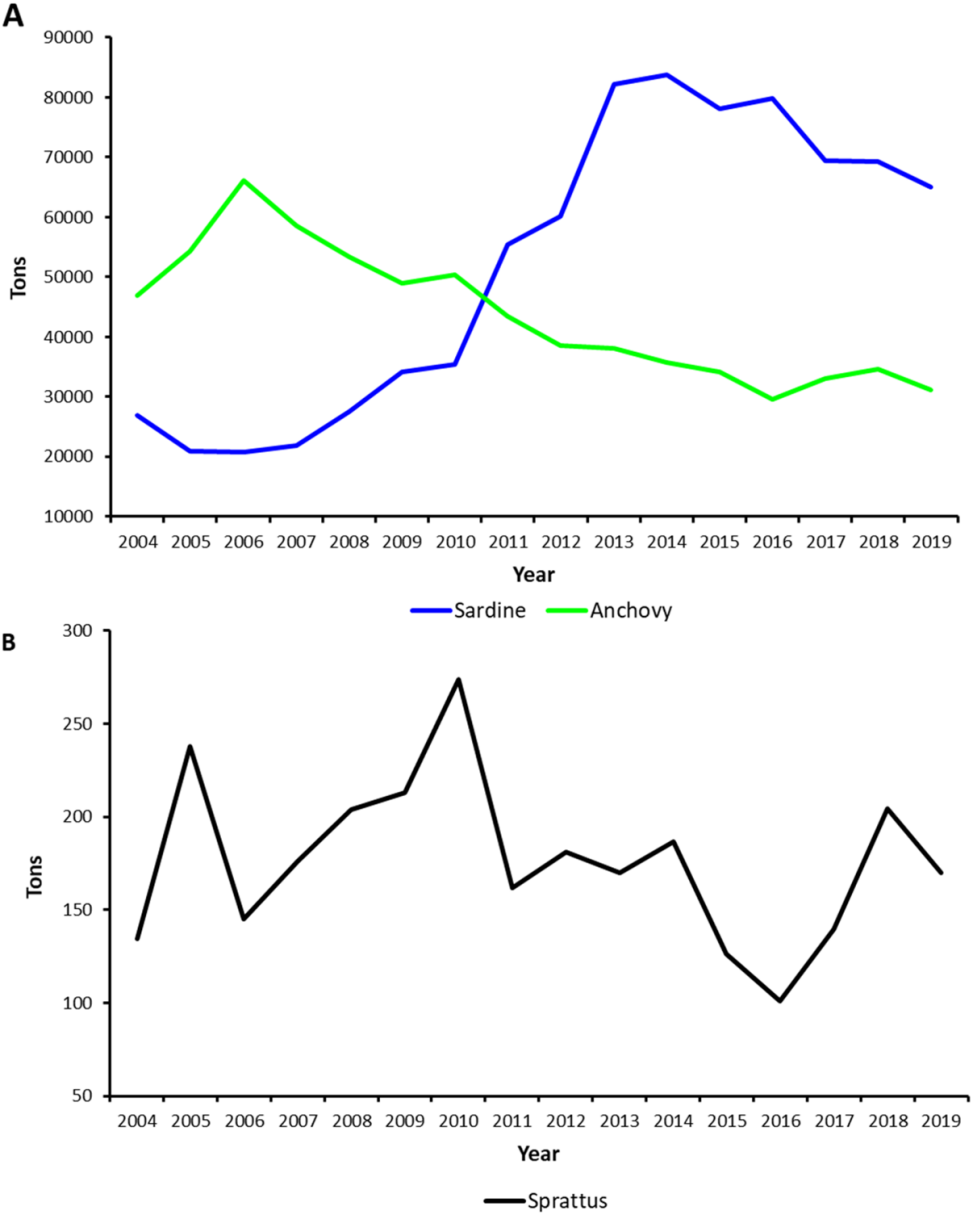
Landings data for (**A**) anchovy (green) and sardine (blue) and (**B**) sprat (black) expressed as tons. Data for anchovies and sardines come from FAO Stock Assessment Forms^5,6^, while data for sprats were taken from Angelini et al.^7^.

**Figure 3:**
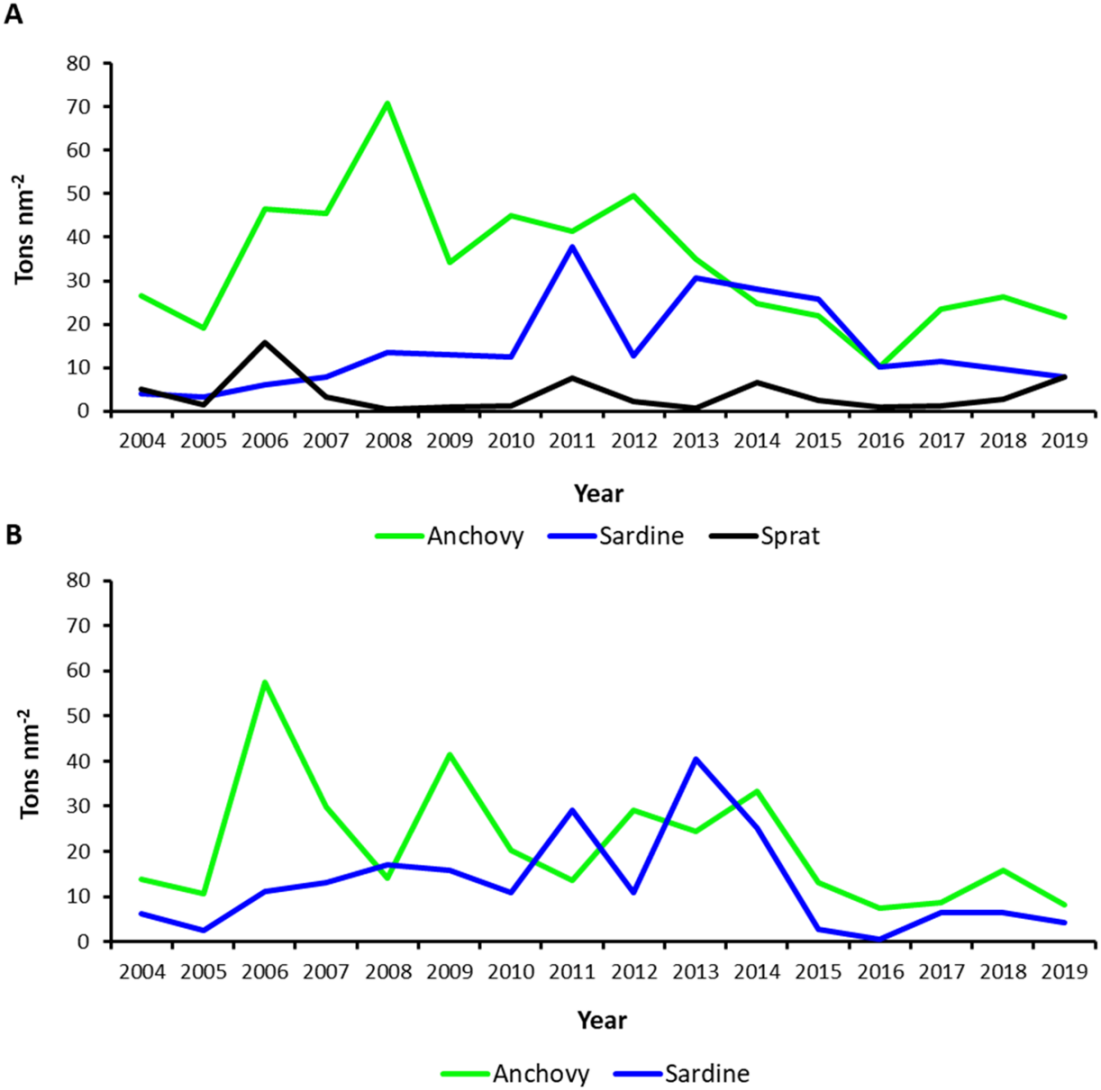
Biomass data expressed as tons per nm^2^ for anchovy (green), sardine (blue) and sprat (black) in (**A**) GSA 17 West and (**B**) GSA18 West. Data were adapted from Leonori et al.^8^.

In the Mediterranean basin, catches of anchovies and sardines represent the 52.5% of total landings^3^. Sprats instead play a minor role, being important only for the Northern Adriatic and the Gulf of Lions^2,4^. Despite anchovies and sardines are managed by the General Fisheries Commission for the Mediterranean (GFCM) through multiannual plans in the Adriatic Sea, small pelagic fish stocks can experience strong fluctuations that could make it difficult to keep the fishing effort at sustainable levels. Indeed, as suggested by the most recent stock assessments^5,6^, both anchovies and sardines are currently overfished in the Adriatic Sea, with a more severe toll on sardines (Fig. 2A). On the other hand, sprat has very limited landings, and according to an experimental stock assessment for 2018^7^ is quite underexploited (Fig. 2B and Table 1). The analysis of long-term data series^8^ showed an increase of anchovies in the Northwestern Adriatic Sea (Geographical Sub-Area, GSA 17, data from 1976 to 2019) and a quite stable trend in the Southern Adriatic Sea (GSA 18, data from 1987 to 2019), while both sardines and sprats decreased in the whole basin (Fig. 3). Opposite biomass contractions observed between anchovies and sardines in the Adriatic Sea in the last 40 years were attributed to regime shifts^9^.

**Table 1:** Reference points obtained from recent stock assessments for sprat, sardine, and anchovy.

Sprat						
Year	F_msy_	F_cur_	F_cur/Fmsy_			
2018	0.30	0.11	0.37			

Sardine						
Year	F_msy_	F_cur_	F_cur/Fmsy_	B_lim_	B_cur_	B_cur/Blim_
2014	0.72	1.09	1.52	125,318	208,604	1.664597
2015	0.72	1.49	2.08	125,318	183,873	1.467251
2016	0.72	1.30	1.82	125,318	161,297	1.287102
2018	0.44	1.53	3.48	125,318	157,251	1.254816
2019	0.47	2.08	4.43	178,200	198,600	1.114478

Anchovy						
Year	F_msy_	F_cur_	F_cur/Fmsy_	B_lim_	B_cur_	B_cur/Blim_
2014	0.55	0.99	1.79	45,936	89,501	1.948385
2015	0.55	0.99	1.79	45,936	86,595	1.885123
2016	0.64	1.43	2.23	45,936	57,469	1.251067
2018	0.57	1.08	1.89	45,936	113,353	2.467629
2019	0.81	1.22	1.51	16,200	17,089	1.054877

Data for anchovy and sardine come from Stock Assessment Forms for GSA 17 and 18 between 2014 and 2019 (https://www.fao.org/gfcm/data/safs/fr), while sprat data were obtained from^7^. *F*_*msy*_ fishing mortality at Maximum Sustainable Yield, *F*_*cur*_ current fishing mortality, *B*_*lim*_ spawing stock biomass limit reference point, *B*_*curr*_ current spawing stock biomass.

Small pelagics mainly feed on phytoplankton and micro/mesozooplankton^10^, resulting in high abundance especially in nutrient-rich upwelling regions^10^, where their significant abundance and success have been attributed to the flexibility of their feeding behavior^11,12^. A recent study conducted along the Adriatic Sea showed a higher mesozooplankton biomass and abundance in the Northern Adriatic, strongly influenced by Po River inputs, where^13^ the community is dominated by the calanoids *Acartia clausi* and *Oithona similis*, cladocerans (mostly *Evadne spinifera*), copepodites, and gastropod larvae. Conversely, the Central and the Southern Adriatic Sea are characterized by an “oceanic” community, with a higher abundance of typical offshore carnivorous zooplankton such as tunicates, chaetognaths, siphonophores and large copepods such as *Euchaeta* spp.^13^.

Small pelagics are planktivorous species, diurnal predators^14^ and with ontogenetic shift in the diet from copepod developmental stages (eggs, nauplii, meta-nauplii and copepodites) in fish larvae to small copepods in juveniles, while adults may switch from preying on larger copepods and other mesozooplankton, to filter-feeding (see Table S1). Grazing on phytoplankton has been rarely reported in anchovies, and it seems to occur only when mesozooplankton is limiting^15^. On the contrary, phytoplankton appears to be an important food item in sardines and sprats^14–16^, especially with increasing size^14,17,18^. Feeding ecology of anchovy and sardine has been already investigated in the Adriatic through visual identification of gut content. Some of these studies conducted in the eastern basin report a dietary overlap between small pelagic species^18^, while few SIA data from the same area point out to no overlap^19^. Due to their intermediate trophic level, small pelagic fishes can play a crucial role in many ecosystems, exerting a negative top-down control on plankton abundance, but also a positive bottom-up control on top predators. However, since small pelagic populations show extensive fluctuations under intensive exploitation, changes in productivity or climate changes, modifying the structure, and functioning of marine ecosystems, they often exert a less predictable ‘wasp-waist’ control^20,21^. Therefore, characterizing better food webs structure and niche overlap has become particularly important since the rise of ecosystem-based management, which aims to create a sustainable exploitation strategy that protects the ecosystem and the goods it provides.

Recently, most of the studies assessing food-web structure and function of pelagic communities, use stable isotope analyses (SIA)^11,12^, in addition to traditional stomach contents analyses or more advanced genetic tools such as metabarcoding^11^ or Compound Specific-SIA^22^. Stable isotope values increase with trophic level due to selective metabolic fractionation, that leads to a preferential loss of lighter isotopes during respiration (carbon) and excretion (nitrogen). Nitrogen *δ*^15^N and carbon *δ*^13^C are the most common isotopes measured in trophic ecology studies^23^. The increase of *δ*^15^N averages about 3‰ per trophic level, so it can be used to determine the trophic position of the consumers along the food web^24,25^. On the contrary, *δ*^13^C increases below 1‰ per trophic step, and can be used to track the origin of organic matter (pelagic *vs*. benthic, terrigenous *vs*. marine)^24,25^. Stable isotope analyses give a time-integrated picture of fish diet and allow to evaluate the relative contribution of various food sources in consumer’s diet, intraspecific trophic relationships as a response to ontogeny, neighboring-linked connectivity, migration, reproduction, and changes in environmental features^26–28^. However, SIA alone also fails to differentiate between two food sources with overlapping isotope values and among ecological niches of species that have the same isotopic niche^29^. This can be partially solved through Bayesian mixing models to estimate errors concerning turnover rates and isotope ratios of putative food sources^30^.

In this study, using SIA and literature data on the well-known diets of sardines and anchovies and new original data on stomach contents for sprats, we deepen our knowledge about the feeding ecology and the trophic niche overlap among these three species across the Western Adriatic Sea, the most productive and exploited basin within the Mediterranean^31^. Stomach content analysis is a well-known method to study the trophic ecology of fish and provides a snapshot of the diet^32^ but does not allow to identify high-digestible prey and to understand the real proportion of assimilated prey.

More precisely, in this study we aimed at (1) assessing changes in feeding habits of the three species from the northern to the southern Adriatic basin, also considering variations in the diet with increasing size; (2) analyzing resource partitioning and trophic niche overlap; and (3) relating these changes and patterns with resource availability and variations of environmental variables. Finally, as anchovies and sardines are widely distributed in both coastal and offshore waters, while sprats are confined to the neritic zone^33^, for the two former species we also tested the hypothesis of 4) trophic changes across an inshore-offshore gradient.

## Results

### Diet composition of *Sprattus sprattus* based on stomach content analysis

Fifty-eight specimens were individually analyzed to depict sprat’s diet in the area. This sample size was considered sufficient according to the cumulative curve analysis (Fig. S1), as the asymptote was reached at 22 stomachs (containing 18 different items). Sprats mostly fed on small copepods (*Microcalanus* sp., *Calanus* sp., and *Acartia clausi*) and cladocerans (Table S2a), and changes in the diet according to size were just below the level of significance (*pseudo-F*_*1,56*_ = 2.29, p = 0.04). Indeed, SIMPER test did not evidence differences in most typifying species according to size, being *Microcalanus* sp., *Calanus* sp., cladocerans and *Acartia* sp. the taxa that most contributed to sprats’ diet in both medium and large specimens (Table S2b). The stomach fullness (*F*) of sprats was greater for medium-sized specimens from both the Northern Adriatic (NA) and the Southern Adriatic (SA) (*F* = 1.90 ± 0.49 for NA and *F* = 2.18 ± 0.73 for SA, respectively) than for large specimens (*F* = 1.47 ± 0.39, only available from NA).

### Stable isotopes composition of small pelagics

*δ*^15^N values of the 81 sprats analyzed varied from 8.4 to 11.3‰ (mean value 9.7 ± 0.6‰) and *δ*^13^C values from − 20.8 to − 18.1‰ (mean value − 19.8 ± 0.6‰). A total of 189 anchovies were analyzed, with *δ*^15^N values ranging from 6.6 to 12.0‰, (mean value of 9.1 ± 1.3‰) and *δ*^13^C from − 20.9 to − 18.1‰ (mean value = − 19.0 ± 0.5‰). For sardines, 138 specimens were analyzed, with *δ*^15^N values ranging from 6.3 to 10.9‰ (mean value 8.91 ± 0.9‰) and *δ*^13^C from − 22.4 to − 18.7‰ (mean value = − 19.9 ± 0.8‰) (Table 2).

**Table 2:** (a) Mean values of *δ*^15^N, *δ*^13^C, C/N (± sd, standard deviation) and trophic position (TP) obtained from stable isotope values (TP SIA) and from Fishbase (TP FB, www.fishbase.se accessed on July 2023) of the specimens of *Sprattus sprattus*, *Sardina pilchardus* and *Engraulis encrasicolus* collected in North, Central and South Adriatic Sea; (b) mean values of *δ*^15^N, *δ*^13^C (± sd, standard deviation) and trophic position (TP) obtained from stable isotope values (TP SIA, based on a fixed TEF of 3.3) and from Fishbase (TP FB, www.fishbase.se accessed on July 2023) of the specimens of *Scomber scomber*, *S. colias*, *Trachurus mediterraneus*, *T. trachurus*, *Euthynnus alletteratus*, *Thunnus thynnus*, *Sarda sarda*, *Xiphias gladius*, from our own data and from literature.

(a)	*δ*^15^N (‰)		*δ*^13^C (‰)		C/N		TP	TP
	Mean	s.d	Mean	s.d	Mean	s.d	SIA	FB
North Adriatic								
*S. sprattus*	9.7	0.6	− 19.9	0.5	4.5	1.2	3.8	3.1
*S. pilchardus*	9.1	0.9	− 20.0	0.8	2.8	0.2	3.7	3.1
*E. encrasicolus*	9.8	1.2	− 19.1	0.7	2.4	0.3	3.8	3.4
Central Adriatic								
*S. pilchardus*	8.8	1.0	− 19.7	0.7	2.5	0.4	3.6	3.1
*E. encrasicolus*	9.0	1.3	− 19.0	0.4	2.8	0.2	3.6	3.4
South Adriatic								
*S. sprattus*	8.9	0.3	− 18.8	0.5	15.9	0.5	3.9	3.1
*S. pilchardus*	8.4	0.8	− 19.5	0.8	2.3	0.2	3.4	3.1
*E. encrasicolus*	8.2	0.9	− 18.9	0.4	2.7	0.2	3.3	3.4

(b) Species	δ^15^N	δ^13^C	TP SIA	TP FB	Source			
*Scomber scombrus*	11.1 ± 0.7	− 18.95 ± 0.3	4.2	3.5	Da Ros et al.^29^			
*Scomber colias*	9.1 ± 0.5	− 18.40 ± 0.2	3.6	3.8	Da Ros et al.^29^			
*Trachurus mediterraneus*	9.7 ± 0.5	− 18.57 ± 0.5	4.0	3.8	Da Ros et al.^29^			
*Trachurus trachurus*	9.4 ± 0.6	− 19.27 ± 0.1	3.7	4.0	Da Ros et al.^29^			
*Euthynnus alletteratus*	11.1 ± 0.4	− 17.03 ± 1.0	4.4	4.4	Authors’ unpubl. data			
*Thunnus thynnus*	12.4 ± 0.7	− 17.4 ± 0.5	4.6	4.4	Authors’ unpubl., Sarà and Sarà^34^			
*Sarda sarda*	10.3 ± 0.8	− 17.6 ± 0.7	4.2	4.5	Authors’ unpubl			
*Xiphias gladius*	12.9 ± 0.4	− 18.4 ± 0.9	5.0	4.05	Authors’ unpubl			
*Tursiops truncatus*	15.6 ± 2.1	− 16.5 ± 1.2	5.6		Fortibuoni et al.^35^			

**Figure 4:**
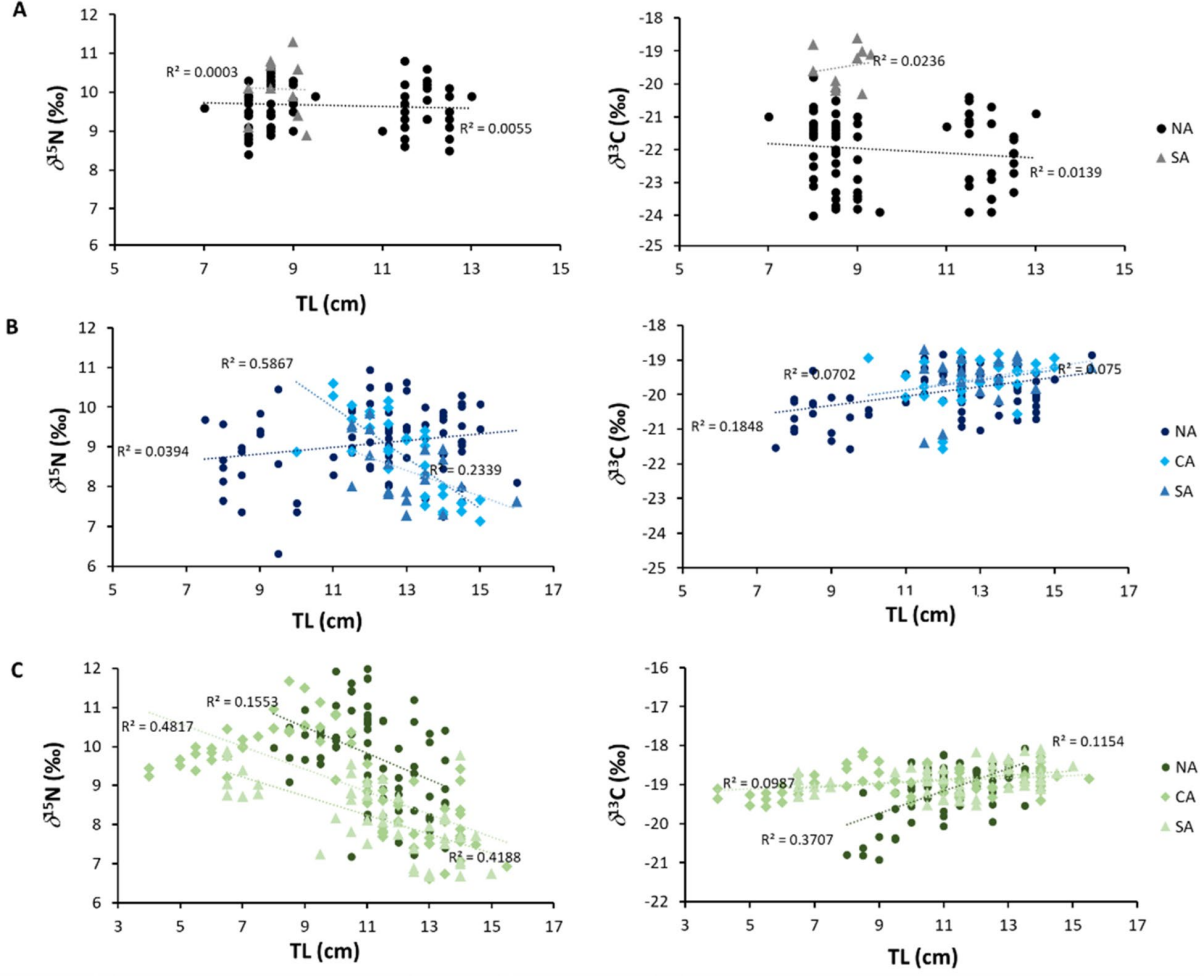
*δ*^15^N *vs* TL (Total Length) (left) and *δ*^13^C *vs* TL (right) scatterplots of (**A**) *Sprattus sprattus*, (**B**) *Sardina pilchardus* and (**C**) *Engraulis encrasicolus,* with the polynomial or linear relationship reported with fish length for anchovy, sardine and sprat in the NA (North Adriatic), CA (Central Adriatic) and SA (South Adriatic) sub areas.

The Pearson correlation of *δ*^15^N to fish total length (TL) is non-significant (p > 0.05) for sardines, and significant for sprats and anchovies (p < 0.001) (positive and negative correlations, respectively) (Fig. 4 and Table 3). *δ*^13^C was significantly (p < 0.001) and negatively correlated with TL in sprat, while correlations were positive and significant (p < 0.001) for sardine and anchovy (Fig. 4 and Table 3). Correlation values for each species at sub-area level are reported in Table S3. The correlation of *δ*^15^N *vs*. fish length was never significant for sprat. For sardines, correlation was negative and significant in CA and in the Southern Adriatic Sea (SA), and it was always negative and significant for anchovies in all sub-basins. *δ*^13^C–TL correlations were positive and significant in Northern Adriatic Sea (NA) for sardines, and positive and always significant for anchovies.

**Table 3:** Results of the correlation analysis between *δ*^15^N and *δ*^13^C *vs*. TL, Pearson R and p-values are reported.

	*δ*^15^N-TL		*δ*^13^C-TL	
	R	p	R	p
*S. sprattus*	0.41	0.0003	− 0.49	0.0001
*S. pilchardus*	− 0.08	n.s.	0.33	0.0004
*E. encrasicolus*	− 0.50	0.0001	0.33	0.0001

*n.s.* not significant.

To comply with the aims of the study, we carried out statistical analyses to identify the resource partitioning among specimens of different species and size. The isotopic composition of the three species varied significantly for the factors ‘species’, ‘size’, and their interactions according to the PERMANOVA main tests at both multivariate and univariate levels (Table S4a, design 1). Pairwise comparison on the interaction term for pairs of levels of factor species showed significant differences between all pairs considered for both medium and large specimens, with the only exceptions of the pairwise on *δ*^15^N values between medium-sized sprat and anchovy and on *δ*^15^N values between large-sized sardine and anchovy (Table S4b). Similarly, when examining differences of size within the same species, all combinations were significant, i.e., for all species the isotopic composition between medium and large-sized individuals varied significantly, at multivariate or univariate level, with the only exception of *δ*^15^N values in sprats and sardines (medium and large specimens) (Table S4c).

Still the isotopic composition of small pelagics varied among the three species in the different sub-basins (Table S4a, design 2). Within NA, significant variations were detected between all pairs of species, except for *δ*^15^N values of anchovy and sprat (Table S5b), and the *δ*^13^C values of sardine and sprat. In CA, significant variations were detected between anchovy and sardine, except for *δ*^15^N values. In SA, all pairs of comparisons were significant apart the *δ*^15^N values of anchovy and sardine and the *δ*^13^C values of anchovy and sprat*.*

Finally, to depict better the resource partitioning among anchovy and sardine across an inshore-offshore gradient, the PERMANOVA main test carried out considering a two-factor design (‘species’ and ‘inshore vs. offshore areas’) highlighted significant differences in the isotopic composition of anchovy and sardine collected in inshore *vs*. offshore areas both at univariate and multivariate levels, but not for the interaction of the term ‘species × inshore *vs.* offshore areas’ at univariate level considering *δ*^13^C contents (Table S6a, design 3). The two species varied significantly for the variable(s) considered in inshore areas (Table S6b), but not for *δ*^15^N in offshore areas. At species level the isotopic composition of both anchovy and sardine differed significantly between inshore and offshore (Table S6c).

### Linking isotope composition with environmental variables and resource availability

Environmental variables drive resources availability for small pelagics. Different resources and environmental variables were found to drive small pelagics’ isotopic signals and thus, the assimilated proportion of food sources. In sprats, the dissolved oxygen concentration was the main explanatory variable, accounting for 20% of the variance (Table 4a). Other environmental variables such as turbidity and salinity added less to the explained variance (1–3%) and they were not significant. Similarly, in sardines, the diet was mainly driven by resource availability, i.e., omnivore zooplankton (both groups, those with preference for carnivory and those for herbivory) which together explained 16% of the total variance. However, also adding the other variables, both environmental (salinity and temperature) and biological (abundance of carnivore zooplankton) the total explained variance was only 22% (Table 4b), being in addition not significant. Finally, the anchovy diet seemed to be mainly controlled by environmental variables (mostly salinity and temperature, but also turbidity, fluorescence, and dissolved oxygen) which together accounted for 49% of the total variance (Table 4c).

**Table 4:** Results of DISTLM models run on SIA data *vs.* environmental variables and resource availability, according to the different trophic guilds for (A) *Sprattus sprattus*, (B) *Sardina pilchardus*, (C) *Engraulis encrasicolus*.

Variable	AIC	Pseudo-F	p	Prop	Cumul	Res.df
(A)						
DO_2_	− 34.6	19.99	0.001	0.20	0.20	79
(B)						
Ab_OMN-CARN	38.75	17.31	0.001	0.11	0.11	136
Ab_OMN-HERB	33.39	7.39	0.003	0.05	0.16	135
S	29.25	6.09	0.002	0.04	0.20	134
T	28.53	2.65	0.07	0.02	0.21	133
Ab_CARN	28.36	2.09	0.14	0.01	0.22	132
(C)						
S	87.99	50.32	0.001	0.21	0.21	187
T	73.08	17.42	0.001	0.07	0.28	186
Ab_CARN	64.50	10.64	0.002	0.04	0.32	185
Turb	44.96	22.22	0.001	0.07	0.39	184
Fluo	35.15	11.80	0.001	0.04	0.43	183
DO_2_	16.36	21.16	0.001	0.06	0.49	182

*Ab_OMN-HERB* Abundance of omnivore-herbivore zooplankton, *Ab_OMN-CARN* Abundance of omnivore-carnivore zooplankton, *Ab_CARN* Abundance of carnivore zooplankton, *Turb* Turbidity, *DO*_*2*_ dissolved oxygen concentration, *S* Salinity, *T* Temperature, *Fluo* Fluorescence, *n.s.* not significant.

### Mixing models and niche width by standard ellipse areas corrected for small sample size (SEA_C_)

**Figure 5:**
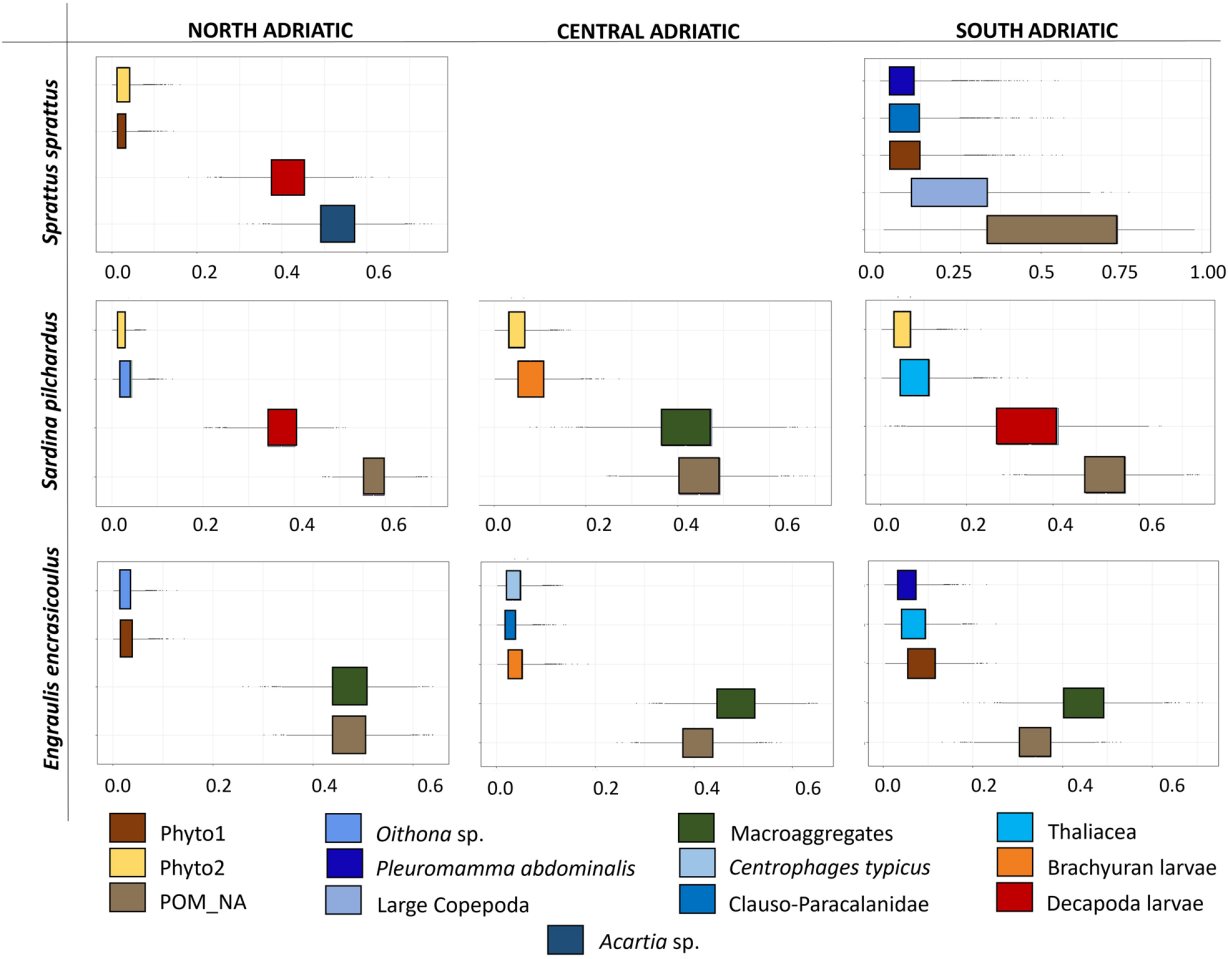
Posterior probabilities for the proportional source contribution to *Sprattus sprattus–Sardina pilchardus–Engraulis encrasicolus* diets from SIMMR output for Northern Adriatic Sea, Central Adriatic Sea and Southern Adriatic Sea. Phyto1 and Phyto2 are the SI values of phytoplankton from Northern Adriatic^36^, POM_NA is the SI of the Particulate Organic Matter (POM) from the Northern Adriatic^37^. Macroaggregates are the stable isotope (SI) signatures of macroaggregates from the Northern Adriatic^36^.

The Bayesian mixing model SIMMR provided the proportional contribution of each food source to the diet of the three species. In NA, the main contribution to the diet of *S. sprattus* was given by *Acartia* sp. and Decapoda larvae (53 and 41% of proportional contribute, respectively). In SA, the main assimilated source was the Particulate Organic Matter (hereafter, POM) (52%), followed by large Copepoda (22%). Minor contributions were given by phytoplankton (9%), Copepoda of the family Clauso-Paracalanidae (9%) and *Pleuromamma abdominalis* (8%) (Fig. 5).

POM gave the main contribution to the diet of sardines in NA (58%), followed by Decapoda larvae (37%). In CA, POM and macroaggregates from NA mostly contributed to the diet of sardines (45 and 42% of proportional contribution, respectively). Minor contribution was given by Brachyuran larvae (8%). In SA, POM gave 52% of the proportional contribution to the diet of sardine, followed by Decapoda larvae that contributed 34%. Minor contribution was given by Thaliacea (9%) (Fig. 5).

In NA, POM and macroaggregates gave the main contribution to the diet of anchovy (47% of the proportional contribute from both the sources). Macroaggregates and POM were the main sources in CA (49 and 40% of proportional contribution, respectively). In SA, macroaggregates contributed to the diet of anchovy for 44% while 34% of contribution derived from POM. Minor contributions were given by phytoplankton (9%), Thaliacea (7%) and *P. abdominalis* (6%) (Fig. 5).

**Figure 6:**
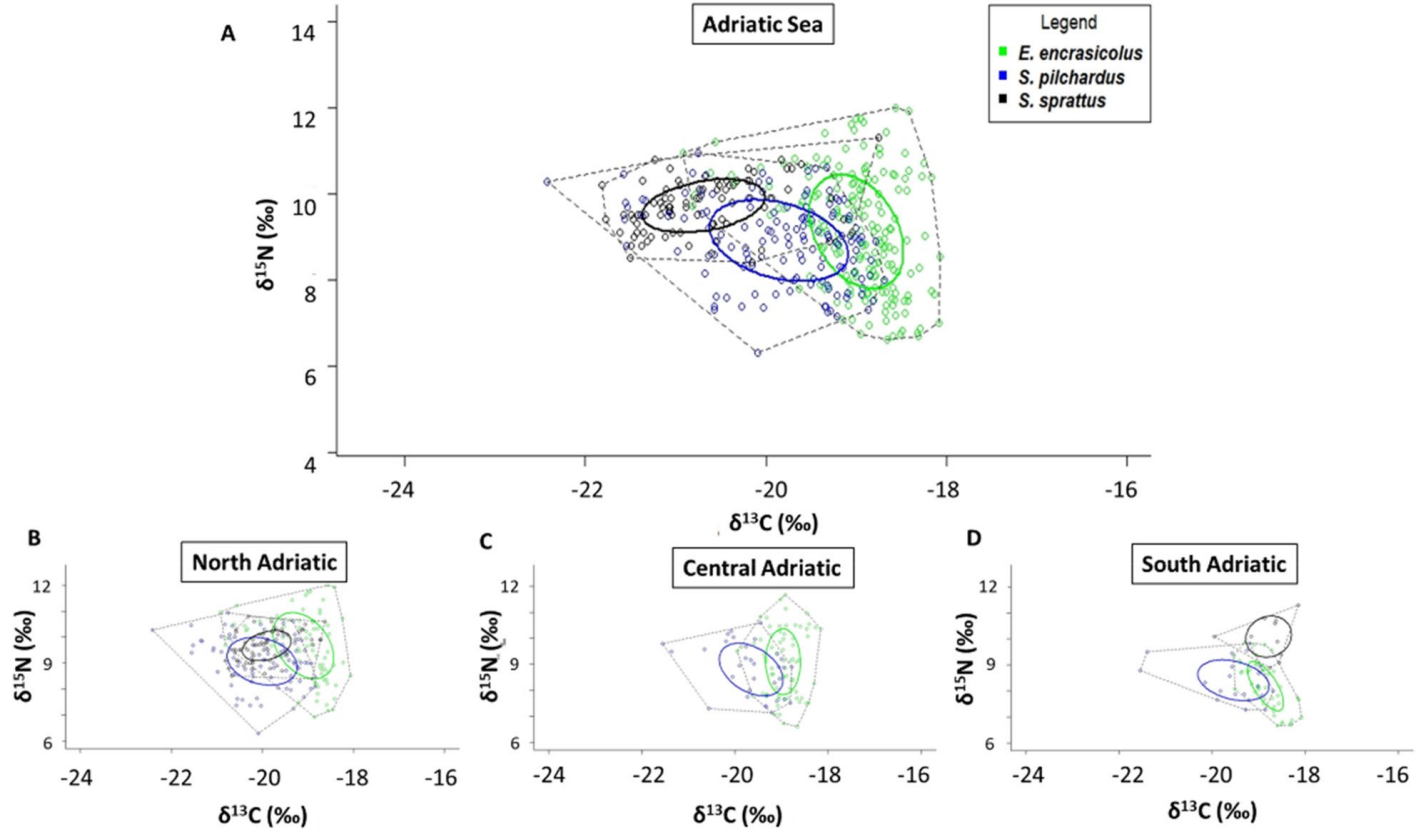
*δ*^13^C–*δ*^15^N scatterplot with total area (TA, broken-line areas) and standard ellipses corrected for small sample size population (SEA_C,_ solid-line areas) of the three species (p interval = 0.4) in (**A**) the Adriatic Sea; (**B**) the North Adriatic Sea; (**C**) the Central Adriatic Sea and (**D**) the South Adriatic Sea.

Standard ellipses showed that sprat has the widest *δ*^13^C variability and anchovy the smallest one (Fig. 6). Anchovy has the widest *δ*^15^N variability. Additionally, Layman metrics indicated that sprat had the smallest SEA_C_ (1.2‰^2^), stretched along the x-axis, while anchovy and sardine showed a similar SEA_C_ (around 2‰^2^), being that of anchovy stretched along the y-axis (Fig. 6 and Table S7). In NA, anchovies show the widest SEA_C_. In CA and SA, sardines have thew widest SEA_C_ (Table S7).

Sprats showed the highest mean distance to the centroid (CD = 0.6 for sprat *vs* CD = 0.54 for anchovy and CD = 0.3 for sardine), which is a proxy of trophic diversity. Sprat and anchovy’s SEA_C_s do not overlap, while a partial overlap is displayed between the SEA_C_s of sardine and anchovy (0.32) and between sprat and sardine (0.23) (Fig. 6).

**Figure 7:**
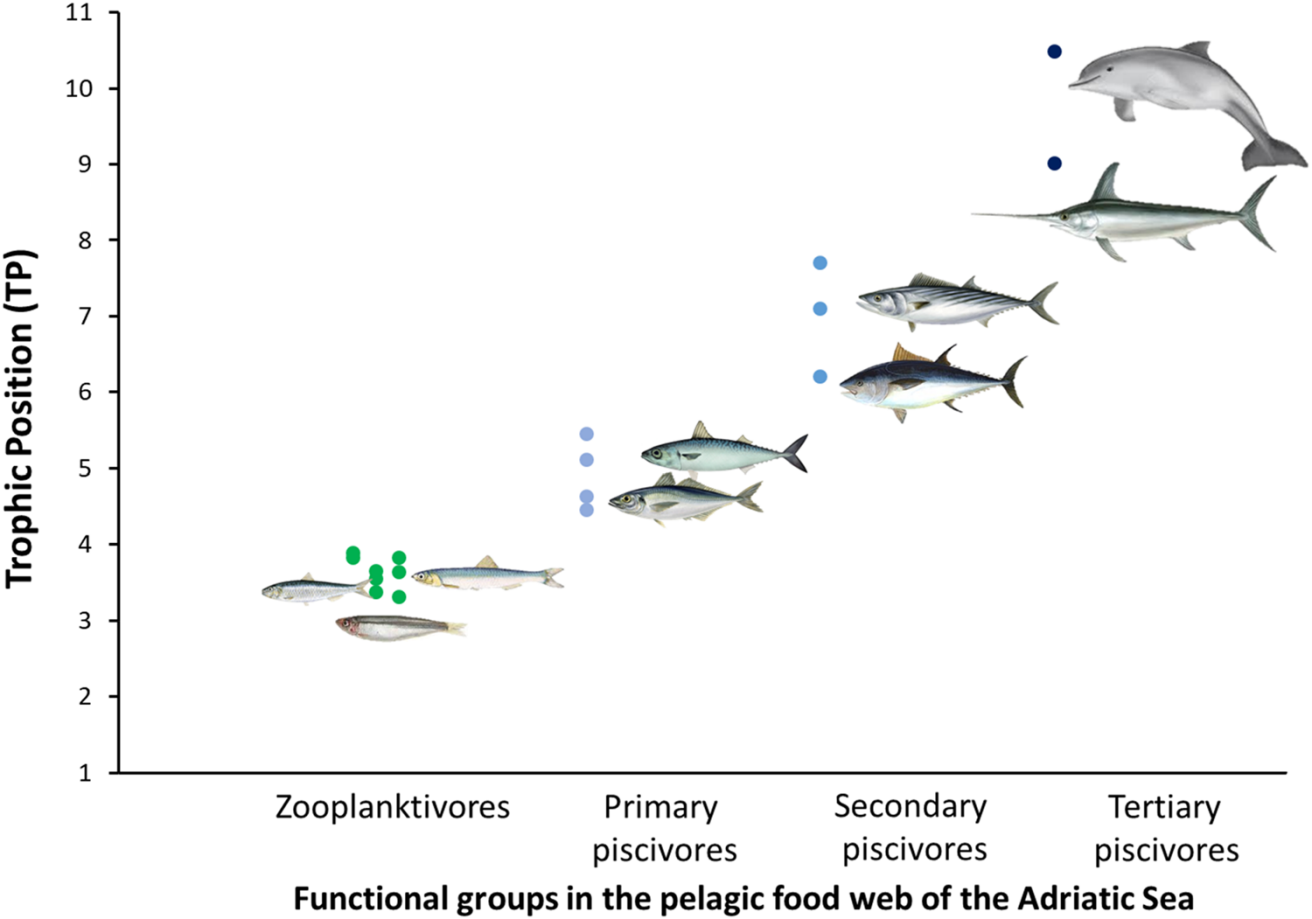
Scatterplot of isotopic data for zooplankton, sprat, sardine, anchovy, large pelagic fish and bottlenose dolphin. Data for large pelagics are from Sicilian waters and have been previously corrected with respect to a common baseline (i.e., comparing the stable isotope signature of herbivore-omnivore copepods from Rumolo et al.^38^ with our own data) and specifically, *δ*^13^C–*δ*^15^N values for *Thunnus thynnus* are from Sarà and Sarà^34^ and are related to specimens of 95–130 cm TL; data for *Euthynnus alletteratus*, *Sarda sarda* and *Xiphias gladius* are from Fanelli unpublished data and corresponds to specimens of the following dimensions: *E. alletteratus* 39.5–41 cm TL; *S. sarda*: 55–58 cm TL, and *X. gladius*: 200–203 cm Fork length. Data for *Tursiops truncatus* are from Fortibuoni et al.^35^. *Tursiops truncatus* was redrawn from https://oceanomaredelphis.org/bottlenose-dolphin/. All the fishes were redrawn from images available at: https://en.wikipedia.org/wiki/Atlantic_bluefin_tuna#/media/File:Bluefin-big.jpg; https://it.wikipedia.org/wiki/Engraulis#/media/File:Engraulis_encrasicolus_Gervais_flipped.jpg; https://species.wikimedia.org/wiki/Sardina_pilchardus#/media/File:Sardina_pilchardus_Gervais.jpg; https://it.wikipedia.org/wiki/Sprattus_sprattus#/media/File:Sprattus_sprattus_Gervais.jpg; https://en.wikipedia.org/wiki/Atlantic_bonito#/media/File:Sarda_sarda.jpg; https://it.wikipedia.org/wiki/Euthynnus_alletteratus#/media/File:Euall_u0.gif; https://it.wikipedia.org/wiki/Xiphias_gladius#/media/File:Xiphias_gladius1.jpg; https://arrankoba.com/wp-content/uploads/2022/06/trachurus_trachurus.jpg; https://www.colapisci.it/PescItalia/pisces/Carangiformes/Carangidae/maggiore/pic/Trachurus%20mediterraneus-fr.jpg; https://www.fisheries.noaa.gov/s3/2021-04/640x427-Atlantic-chub-mackerel-FNL_NB_W.jpg; https://it.wikipedia.org/wiki/Scomber#/media/File:Scomber_scombrus.png.

According to the Post equation for the trophic position (TP) estimation, the three small pelagics were all positioned at the third trophic level, with TPs ranging from 3.3 in *E. encrasicolus* from SA to 3.9 in *S. sprattus* from the same area. The food web of the Adriatic Sea seemed to be better represented by a continuum of trophic levels rather than discrete ones (see Fig. 7) with “ancillary” small pelagics^29^, located at the TP 4, followed by small tunnids^34^, and potentially preyed by large pelagic species such as the swordfish, *Xiphias gladius* (authors’ unpublished data), and the bottlenose dolphin, *Tursiops truncatus*, the apex predators of the pelagic food web of the Western Adriatic Sea^35^.

## Discussion

Our study allowed to assess the resource partitioning and trophic niche overlap among sprat *Sprattus sprattus*, sardine *Sardina pilchardus* and anchovy *Engraulis encrasicolus* in different sub-areas of the Adriatic Sea and at different life stages. The results of the study emphasize the feeding plasticity of these pelagic species as previously observed for anchovy in the Adriatic Sea^39^, and in other areas of the Mediterranean such as the Gulf of Lions^40^ and the Sicily Strait^16^.

Our results allowed us to define the trophic habits of sprat in the western side of the Adriatic Sea. Here, sprats mainly feed on calanoid copepods of the genus *Microcalanus* and *Calanus*, two taxa that characterized more than half of the diet of this species in both the Northern and Southern Adriatic. However, it seems that these taxa are an important food source even if they are not the most abundant during the sampling period^13^, highlighting a specialist feeding behavior of sprats^41–44^. These SCA results are consistent with those of previous studies^41^. The species seemed to have a quite constant diet as demonstrated in other studies conducted in the Black Sea^42^, in the Gulf of Lions^43^ and in the Bay of Biscay^44^.

Concerning SIA data, sprats showed an increase of *δ*^15^N values with increasing total length, pointing out to an ontogenetic shift in the diet as observed with SCA for sardine and anchovy of Algerian waters and the Northern Adriatic^39,45^. Ontogenetic shifts in a species’ trophic level reduce intra-competition for feeding and probably allows larger individuals to sustain better energy consumption facing the spawning period. In the Adriatic Sea, sprats spawn between November and April^41^. Larger sprats caught during MEDIAS surveys (June–July) seemed to be specialized in capturing larger prey, probably recovering energies lost during reproductive period, while smaller specimens likely rely more on particulate organic matter or algal material due to a lesser filter-feeding capacity^43^.

Additionally, in the Adriatic Sea, ontogenetic changes in sprats’ diet likely occur thanks to the high availability of food (e.g., zooplankton abundance^13^), as also observed in another highly productive area, the North Chilean Patagonia, where a similar *δ*^15^N pattern was reported for *Sprattus fuegensis*^46^, suggesting that high primary productivity supports in turn high food availability and, thus, allows to larger and smaller sprat specimens to avoid intra-specific overlapping of trophic niches with a more selective feeding compared to that in areas with lower food availability^46^. On the other hand, the strong decrease in *δ*^13^C with increasing size suggests that in the northern Adriatic larger animals move more inshore, reaching their coastal spawning grounds^41^. The lower *δ*^13^C values of coastal areas in the Western Adriatic Sea is due to the freshwater inputs by the Po River discharge, as observed in other Mediterranean areas^47^.

Conversely, the *δ*^15^N trend of sardine and anchovy best fitted with a polynomial distribution, thus suggesting an ontogenetic shift in their trophic habits as already observed for anchovy in Northern Adriatic^39^ and for sardine in the Algerian Sea^39,45^, and in Galicia^48^. Here, this was interpreted as a dietary shift to phytoplankton consumption in larger fishes, thanks to the acquisition of filter feeding ability following the gill rakers development^49^. The consumption on a wide array of diatoms was recently revealed for species collected in the western Mediterranean through metabarcoding^11^. The increasing trend of *δ*^13^C with size in both species pointed out to an inshore-offshore displacement with growth^43,50^. Such offshore movements to more oligotrophic areas, with marine phytoplankton being the main carbon source, was already observed for sardines in the Gulf of Lions^43^. Although anchovy is generally considered to be more zoophagous than sardine^14,50^, our results are quite unexpected as large specimens of anchovy in the Adriatic Sea seemed to prefer to assume phytoplankton, a feeding behavior that could be validated in the future through metabarcoding^11^. This trend was observed in the co-generic *Engraulis capensis* in South Africa^51^, suggesting also for this species a shift from zooplankton to phytoplankton assumption according to resource availability^38^, but also to the acquisition of filter feeding ability. This preference for phytoplankton could be also supported by the declining trend in mesozooplankton observed since 2000 in the Northern and Central Adriatic (authors’ unpublished data).

Small pelagics have high turn-over rates and short life history^52^ and although any experimental study is available for these or similar species, we can assume the incorporation rate is at the scale of days/weeks and not months, as for larger and slow-growth species^53^. In this study, we used environmental and biological variables collected simultaneously to fish samples, thus introducing potential bias due to the isotopic incorporation rate of the species, i.e., the time required by an organism to acquire the isotopic composition of its new diet^54^, which can be highly variable depending on a species turn-over rate, environmental conditions, the physiological state of the animal, etc.^55^. The variables here used, although with some caveats, can mirror the situation of few weeks before, considering that the typical summer conditions have already been established during the time of the sampling (end of June–July)^56^.

The analyses conducted in our study showed that the *δ*^15^N and *δ*^13^C content of sprat was mainly dependent on water oxygen concentration, which in turn is one of the limiting factors for the survival and the growth of zooplankton, and one of the driving factors of zooplanktonic communities' composition throughout the Adriatic Sea^13^. The isotopic signals of sardines were mostly driven by resource availability rather than environmental variables, which were found instead determinant for larval fish survival^57^, and fish population dynamics^58^. Such results are in contrast with observations on sardines from the Bay of Biscay^44,50^ where no clear link was found between food resource availability and fish diets. In the Adriatic the temporal fluctuations in the abundance of sprats and sardines could be driven by food availability. Therefore, being their abundance dependent to variations in mesozooplankton communities, the monitoring of this last should be essential for the appropriate management of the two species. Indeed, sprats and sardines showed a declining trend in the last 40 years^8^ in agreement with the general decline in zooplankton abundance described above (Authors’ unpubl. data). On the other hand, in anchovy, the *δ*^15^N and *δ*^13^C content was mostly controlled by environmental variables such as salinity and temperature, and the species showed an increasing trend in the abundance in the last 40 years in the Northern-central Adriatic Sea^8^. Thus, for this species climatic factors control its abundance’s fluctuations, rather than its trophic ecology.

Even if for sardine and anchovy further studies are needed to couple SIA with other analyses (e.g., visual stomach content characterization, DNA metabarcoding etc.) to define better their diets, especially for the microscopic components, such as diatoms, the Bayesian mixing model SIMMR allowed us to determine the food sources that were mainly assimilated in the tissues of the three species. The obtained results highlighted that in the Northern Adriatic, sprat preferentially assimilates zooplanktonic items like *Acartia* sp., and Decapoda larvae belonging to higher trophic levels. In Southern Adriatic Sea, sprat prefers to assimilate macroaggregates of Particulate Organic Carbon (POC) and POM, but still feeds on copepods. POC and POM are easily assumed, so probably in the more oligotrophic Southern Adriatic, sprat does not invest too much energy in catching calanoid copepods. The species here is at the southernmost boundary of its distribution^33^, and moreover this is an ultra-oligotrophic area, so on one hand the species could rely preferentially on easy-to-find resources, investing less energy in an unsuitable area, and on the other hand here zooplankton abundance is lower. The same trend could be highlighted for sardines that in all the sub-areas mainly assimilate POM, followed by Decapoda larvae in the North and South Adriatic Sea and phytoplanktonic macroaggregates in the Central Adriatic. Anchovy seemed to mainly assume POM and macroaggregates in all the sub-basins. This species is usually more zoophagous than sardine, but our results demonstrate that in the Adriatic Sea, at least in early summer, anchovies mostly rely on phytoplankton like previously observed in other studies^59^. Data about *δ*^15^N and *δ*^13^C contents showed how these species share a similar trophic position, based on comparable *δ*^15^N values, with some degrees of separation in *δ*^13^C values, meaning that they minimize dietary overlap by recurring to different carbon sources^43^. Accordingly, the three species have similar niche’s width and SEA_C_, but very different *δ*^15^N and *δ*^13^C ranges, confirming that anchovy is the species with the widest *δ*^15^N range and sprat that with the greatest diversity of basal resources. Through this differentiation of *δ*^13^C values of basal resources, trophic niches of the three species in the Adriatic Sea do not overlap. A higher diet overlap was instead observed in the Gulf of Lions^43^, and in the Spanish Mediterranean for anchovy, sardine and round sardinella^60^. The high productivity of the Adriatic basin likely determines a good resource partitioning, as enough food is available for the species to achieve their optimum fitness^44^. As suggested for the Gulf of Lions, the increase of sea surface temperature in the Adriatic basin^61^ and the whole Mediterranean Sea^62^ is expected to drive changes in distribution and increase in the competition for food among small pelagics^63^.

Furthermore, the combined scatterplot of *δ*^15^N and *δ*^13^C values of small pelagic fishes with those of zooplankton, large pelagic fishes, and dolphins (i.e., *Tursiops truncatus*) allowed to stress the central role of anchovies, sardines, and sprats in pelagic ecosystems, being located between zooplankton and larger predators. However, this central role seems to be shared with the so called “ancillary” small pelagics such as *Trachurus* spp. and *Scomber* spp.^29^. Such food web structure could drastically change in a climate change scenario with persistent high level of fishing pressures^64^. Small pelagics are indeed sensitive to environmental fluctuations^63^, and this could be amplified by extreme meteorological events, with cascading effects up and down the food web^65^. Further, the northward expansion of temperate species observed in different areas of the Mediterranean Sea, such as round sardinella^33^ that is able to effectively adapt its feeding activity switching to filter feeding when its preferred food source (i.e., the krill) is scarce, or obtaining their ingested biomass from other large prey like jellyfish and siphonophores as they increase in abundance^60^, may lead to stronger inter-specific competition.

Since sardine and sprat showed a decreasing trend in their abundances across the last 40 years, which could be influenced also by resource availability, a regular monitoring of their resources (i.e., mesozooplankton) should have been done consistently for a correct management of such resources within the ecosystem approach to fisheries. Based on our results, the species with the most adaptative diet in the Adriatic Sea seems to be anchovy that, in all the sub-basins, are likely able to feed on prey belonging to a wider range of trophic levels, thus allowing to encompass diets shifts due to changes in zooplankton community composition caused by climate changes.

## Materials and methods

### Ethical statement

Ethical review and approval were waived for this study, due to fish specimens’ collection being authorized by the MEDIAS project as part of annual research surveys under the EU Fisheries Data Collection Framework (EC 665/2008), all involving lethal sampling. The procedures we used did not include animal experimentation. The care and use of collected animals complied with animal welfare guidelines, laws and regulations set by the Italian Government.

We confirm the study is reported in accordance with ARRIVE guidelines.

### Study area

The Adriatic Sea is an elongated semi-enclosed basin, with its major axis in the northwest–southeast direction, located in the central Mediterranean, between the Italian peninsula and the Balkans (Fig. 1A). It is 800 km long and 150–200 km wide. The North Adriatic is very shallow, with an average bottom depth of 35 m and a maximum depth of 70 m. The Middle Adriatic has a maximum depth of 100 m, except for the Jabuka-Pomo Pit (maximum depth 280 m). In these two areas the eastern part has deeper bottoms, with high and rocky shores, while the western part is shallower, with low sandy shores^56,66^. Despite the differences, these areas share many bioecological features, and for stock assessment purpose they were grouped as GSA 17, according to the GFCM. The southern part of the Adriatic Sea, GSA 18, is deeper with a wide depression of 1200 m deep. Here, the Otranto channel, which is 800 m deep, acts as an exchange area for water masses with the Mediterranean Sea^56,66^.

Despite being only the 5% of the total Mediterranean surface area, the Adriatic Sea produces about 15% of total Mediterranean landings (and 53–54% of Italian landings), with a fish production density of 1.5 t/km^2^, three times the Mediterranean estimate^66^. Three main factors are responsible of such impressive productivity feature: river runoff, shallow depths, and oceanographic structure. By providing nutrients, rivers inputs favor phytoplanktonic blooms, thus causing a bottom-up effect through the whole food web. The wide extension of the continental shelf, together with high environmental variability, favors a short trophic chain^67^, that improves the efficiency of energy transfer from lower trophic levels to higher ones^68^. Moreover, the structure of the basin allows water to be mixed during winter, especially in the northern and central Adriatic, transferring nutrients from sediments to the water column. However, the same condition can be responsible of water stratification, harmful algal blooms, mucilage, dystrophy and anoxia phenomena during summer^66^.

### Sampling strategy

Samples were collected on board R/V “G. Dallaporta” in June–July 2019, during the acoustic survey MEDIAS 2019, in the Adriatic Sea^69^, within the framework of the EU MEDIAS (Mediterranean International Acoustic Surveys) action that coordinates the acoustic surveys performed in the Mediterranean to assess the biomass and spatial distribution of small pelagics in the target areas^8^. According to the MEDIAS protocol^70^ annual hydroacoustic surveys are conducted from June to September. Summer coincides with the peak reproductive period of anchovy and the peak recruitment period of sardine^2,15^.

Simultaneously, pelagic fishes were collected through a pelagic trawl (10 m vertical opening and 12 m horizontal opening, with mesh size 18 mm), equipped with a wireless SIMRAD ITI system that allowed to gather information on the correct opening of the net and on entering fishes during trawling. Hauls of ca. 30 min were performed during both daytime and nighttime, covering as evenly as possible the target area, both inshore and offshore, also considering acoustic data on fish aggregation position in the water column^8^.

Once onboard, fish were sorted, counted, measured (total length—TL—in cm) and weighted (wet weight in g). Since sardines, anchovy and sprats were the main target, 10 individuals per length class (0.5 cm) of each species were frozen at − 20 °C for further laboratory analysis. During the survey, also zooplankton sampling was performed along the acoustic transects, using a 200 µm mesh-size WP2 net, with a circular mouth of 57 cm diameter and 2.6 m long and equipped with a flowmeter. The net was towed vertically, with a towing speed of 1 m/s, starting from 3 m above the bottom till the surface. All samples were sorted, and specimens analyzed to the lowest taxonomic level possible, and the wet weight was calculated. All data were then standardized to the filtered volume of water recorded for each haul. More abundant/representative groups, as resulted after sorting and identifying each sample, were analyzed for determining stable isotopes contents^13^.

After the survey was completed, a set of fishing hauls was selected to be used for this study. The whole Western Adriatic (GSA 17 and GSA 18) has been divided into three different sub-areas^13^, mainly based on oceanographic characteristics: (1) the Northern Adriatic (NA) encompassing the northern part of the GSA 17 characterized by shallow waters, including the Po river mouth, up to the Conero Promontory (hauls 8–24 in Fig. 1A); (2) the Central Adriatic (CA) encompassing the lower part of the GSA 17 up to the Gargano Promontory (hauls 27–39) and (3) the Southern Adriatic (SA) including the whole GSA 18, characterized by the presence of the South Adriatic Pit and the Otranto Channel (hauls 40–46).

In selected hauls, for each species, three individuals per 0.5 bin were chosen for SIA, according to length-frequency distributions (Fig. S2).

### Stomach contents analysis of *Sprattus sprattus*

As information on *S. sprattus* diet from literature was only available for the Eastern Adriatic Sea^41^, to integrate data on SIA for mixing models (see below), the SCA on 58 specimens was carried out. Specimens were thus dissected, and stomach contents identified to the lowest possible taxonomic level. To assess sample size adequacy, the cumulative number of analyzed stomachs was plotted against the mean cumulative number of the different prey species^71^. This was done by using the “species-accumulation” plot in PRIMER6&PERMANOVA+, under 9999 permutations and using bootstrapping. Sample size was considered sufficient when the cumulative prey species curve reached an asymptote, with further changes in the cumulative number of prey items observed < 0.1. The fullness index, as a proxy of feeding intensity, was measured as the ratio of stomach content weight to body weight^28,32,39^.

A PERMANOVA test (Permutational Multivariate Analysis of Variance)^72^ was run only on factor size (fixed, two levels, juveniles and adults) on the Bray–Curtis resemblance matrix of 4th root-transformed biomass (prey weight) data, given the low number of specimens analyzed from the SA. A SIMPER test was also run to identify the most typifying species of the diet of sprat for each size class. All statistical analyses were run using the software PERMANOVA+ for PRIMER^72,73^.

### Samples preparation for stable isotope analyses

For fishes, a small sample of white muscle close to the dorsal fin, from selected specimens (81 sprats ranging from 7 to 13 cm TL, 189 anchovies from 4 to 15.5 cm TL, 138 sardines from 7.5 to 16 cm TL), was oven-dried for 24 h at 60 °C, weighted, between 0.5 and 1.3 mg, and placed into tin capsules, that were then put in a numbered rack. Samples were analyzed through an elemental analyzer (Thermo Flash EA 1112) for the determination of total carbon and nitrogen, and then analyzed for *δ*^13^C and *δ*^15^N in a continuous-flow isotope-ratio mass spectrometer (Thermo Delta Plus XP) at the Laboratory of Stable Isotopes Ecology of the University of Palermo (Italy). Stable isotope ratio was expressed, in relation to international standards (atmospheric N_2_ and PeeDee Belemnite for *δ*^15^N and *δ*^13^C, respectively), as:

$$\delta^{{{13}}} {\text{C }}\;{\text{or}}\;\delta^{{{15}}} {\text{N}}: \, \left[ {\left( {{\text{R}}_{{{\text{sample}}}} /{\text{R}}_{{{\text{standard}}}} } \right) - {1}} \right] \times {1}0^{{3}}$$

where R = ^13^C/^12^C or ^15^N/^14^N. Analytical precision based on standard deviations of internal standards (International Atomic Energy Agency IAEA-CH-6; IAEA-NO-3; IAEA-N-2) ranged from 0.10 to 0.19‰ for *δ*^13^C and 0.02 to 0.08‰ for *δ*^15^N.

Since lipids can alter the values of *δ*^13^C^72^, samples with high lipid concentration can be defatted to avoid ^13^C depletion. However, lipid extraction can alter *δ*^15^N values and thus complicating sample preparation and reducing samples availability, a crucial point when analyzing small animals. For these reasons, *δ*^13^C of samples rich in lipids was normalized according to Post equation^74^ for sardines, anchovies and zooplankton, and to Kiljunen equation^75^ for sprats. C/N ratio was used as a proxy of lipid content, because their values are strongly related in animals^74^. In particular, the normalization was applied to samples with a C/N ratio > 3, according to^74^. Post’s equation was widely used and allowed us to compare our data with other similar studies^12^.

### SIA data analyses

Intra-population variation in the feeding habits explained by body size is a frequent determinant of fish trophodynamics^76^. *δ*^15^N and *δ*^13^C relationships with size (as total length, TL) for the three species, were explored using Pearson correlation, at basin scale and also at sub-area level.

After analyzing the length frequency distribution for each species (Fig. S2), all specimens were assigned to two main size classes, i.e., medium, and large. For *S. sprattus* and *E. encrasicolus* medium-sized individuals were those smaller than 9.5 cm (Total Length, TL), while for *S. pilchardus* the threshold between medium and large was 10.5 cm TL.

According to the objectives, three different experimental designs were used for testing: (i) differences in the isotopic composition of the three species according to the size class (medium *vs*. large), named design 1; (ii). resource partitioning among the three species across the different sub-areas (design 2) and iii. resource partitioning among the anchovy and sardine across an inshore-offshore gradient (design 3), as for mesozooplankton in^13^ (Table 5). This approach was selected because it allowed pairwise comparisons among fixed factors, otherwise impossible with nested designs, due to the unbalanced distribution of the species, i.e., sprat only occurring in NA and SA, and in SA only medium-sized specimens were collected, similarly medium-size sardine only occurred in NA and only inshore at SA.

**Table 5:** Experimental design used to test the three different hypotheses.

Name	Factors	Levels
Design 1	Species	3 (all species)
	Size	2 (medium *vs*. large)
Design 2	Species	3 (all species)
	Sub-basin	3 (NA, CA, SA)
Design 3	Species	2 (*S. pilchardus and E. encrasicolus*)
	Inshore *vs*. offshore	2 (inshore *vs*. offshore stations)

All factors are fixed.

Differences were tested by means of PERMANOVA^72^ tests on the Euclidean resemblance matrix of untransformed univariate (*δ*^15^N or *δ*^13^C, separately) and bivariate (*δ*^15^N–*δ*^13^C) matrices.

### Correlation with biological (zooplankton abundance) and environmental data

To identify the biological (i.e., resource availability) and environmental drivers of the trophic ecology, as by SIA, of the small pelagics in the Adriatic basin, stable isotope data of the three species separately were correlated to zooplankton abundance (i.e., number of taxa recorded at each haul, as detailed in Section 2.2) by trophic group, as by^13^, and to environmental variables, as described below. Environmental data, obtained by CTD casts carried out close to the sampling hauls^13^, were pressure (dbar, decibar), temperature (°C), fluorescence (µg/l), turbidity (NTU), dissolved oxygen (expressed as ml/l and saturation percentage), salinity and density (kg/m^3^). Biological and environmental data were first tested for collinearity among variables by using a Draftsman plot, with fluorescence, Dissolved O_2_ concentration (DO, ml/l), % of O_2_ saturation and turbidity data being Log (x + 1)-transformed to fit a linear distribution in the Draftsman plot. Finally, a DistLM (Distance based linear models^72^) was run with temperature, fluorescence, turbidity, dissolved oxygen and salinity as environmental variables, and the abundances of omnivore-carnivore, omnivore-herbivore and carnivore zooplankton as resource availability, using “step-wise” as selection procedure and “AIC (Akaike Information Criterion)” as selection criterion.

### Bayesian mixing models and trophic level estimates

A Bayesian model with “SIMMR” package (Stable Isotope Mixing Models in R)^77^ was run to estimate the potential food sources for the three species separately under the software R 4.0.5^78^. Before running the model, the isotopic values of the sources and the three fishes were plotted, applying the correct trophic enrichment factors (TEFs) to potential sources to build the mixing polygons^79^. As TEFs, we used a value of 1.3 ± 0.1‰ for *δ*^13^C^80^ and 3.3 ± 0.2‰ for *δ*^15^N^81^. According to^80^, the first value was the best estimate of *δ*^13^C for consumers analyzed as muscle tissue, while the second is the *δ*^15^N specifically estimated for zooplanktivorous species, based on a scaled framework approach^81^. The sources used in the mixing model were selected among those highlighted as dominant in literature (for sardine and anchovy, see Suppl. Table S1) and SCA results (for sprat, see Table S2), and that allowed to construct the best mixing plot^79^. Stable isotope (SI) signatures of the sources used to construct the best mixing polygon were taken from literature (Table S8). We used POM, macroaggregates and phytoplankton isotopic values from the Northern Adriatic Sea to run the mixing models in the three basins, also in the Central and Southern Adriatic, as other values of POM available from literature^82^ did not allow to close the mixing polygons. Furthermore, the use of POM_NA is justifiable because cascading phenomena of dense shelf waters from the Northern Adriatic occur periodically in the area and are found to affect zooplanktonic communities in the Southern basin^83^. The SI signatures of zooplanktonic species were taken from the dataset of a previous study^13^. Macroaggregates are the SI signatures of macroaggregates from the Northern Adriatic^36^, POM_NA is the SI of POM (Particulate Organic Matter) from the Northern Adriatic^37^, Phyto1 and Phyto2 are the SI values of phytoplankton from the Northern Adriatic^36^. Consistently with the best mixing polygons determined for the three species of fishes collected in the different sub-areas (Fig. S3), we used the selected sources to run SIMMR models, specifically, for each of the three sub-areas.

The SIBER package (Stable Isotope Bayesian Ellipses in R 3.5.3)^84^ was then used to calculate TA and SEA_C_ (respectively, Total Convex Hull Area and Standard Ellipse Areas corrected for small sample size)^85^ and standard ellipse areas (p interval = 0.40 to encompass the 40% of our data) for the three species. Moreover, *δ*^15^N range (NR), *δ*^13^C range (CR), and the mean distance to centroid (CD), that is considered a proxy for estimating trophic diversity, were calculated for all the species^84,85^. The overlap of the SEA_C_ of the three different species was calculated through the function “maxLikOverlap” included in SIBER package.

Additionally, the trophic level of the three species in the different sub-areas was estimated according to^24^ as: ((*δ*^15^N_i_ − *δ*^15^N_PC_)/TEF) + λ.

where *δ*^15^N_i_ is the *δ*^15^N value of the taxon considered, *δ*^15^N_PC_ is the *δ*^15^N values of a primary consumer, i.e., an herbivore or a filter feeder, used as baseline of the food web, TEF is the trophic enrichment factor which is considered varying between 2.54^86^ and 3.4^24^, and here is assumed to be 3.3 as above^81^, and λ is the trophic position of the baseline, which is 2 in our case. Here, we used three different values as baselines for the food web of the three sub-areas, specifically the average values of FF-HERB taxa for each sub-area, as from Fanelli et al.^13^.

Finally, the pelagic food web of the Adriatic Sea was depicted by plotting *δ*^13^C and *δ*^15^N mean values of primary producers, mesozooplankton, small pelagics and large pelagic fishes, from our own published^13^ and unpublished data, and from literature^35–37,82^.

### Supplementary Information

Supplementary Information.

## Data Availability

Data can be requested from the corresponding author upon reasonable request.
