# Supplementary material for: The pelagic food web of the Western Adriatic Sea: a focus on the role of small pelagics
Source: Sci Rep. 2023 Sep 4;13:14554. doi: 10.1038/s41598-023-40665-w (PMC10477188; doi:10.1038/s41598-023-40665-w)
Supplement: Supplementary file 1 — Supplementary Information. [file 41598_2023_40665_MOESM1_ESM.docx]

Supplementary Material

**The pelagic food web of the Western Adriatic Sea: a focus on the role of small pelagics**

**Fanelli E.^1,2,3 *^, Da Ros Z.^1^, Menicucci S.^3,4^, Malavolti S.^3^, Biagiotti I.^3^, Canduci G. ^3^, De Felice A.^3^, Leonori I.^3^**

^1^ Department of Life and Environmental Sciences, Polytechnic University of Marche, Via Brecce Bianche 60131, Ancona (Italy)

^2^ Stazione Zoologica Anton Dohrn, Villa Comunale, Naples (Italy)

^3^ CNR-National Research Council, IRBIM-Institute for Marine Biological Resources and Biotechnologies, Largo Fiera della Pesca 6010060125, Ancona (Italy)

^4^ ALMA MATER STUDIORUM, Università di Bologna, Via Zamboni, 33, 40126, Bologna, Italy

*****correspondence:** *Corresponding Author: E. Fanelli,* [*e.fanelli@univpm.it*](mailto:e.fanelli@univpm.it)

**Table S1.** List of the main items found in the diets of *Engraulis encrasicolus*, *Sardina pilchardus* and *Sprattus sprattus* as well as prey size, prey-predator size ratio (PPSR), prey selection (based on Ivlev’s selectivity index), niche width (based on stomach contents) and isotope niche (Standard Ellipse Area, SEA_B_), based on the main recent papers reporting results of studies conducted from 1998 onward in the Mediterranean Sea. The site and sub-basin where the studies were conducted are also reported: NW Med, SW Med, C Med, E Med are Northwestern, Southwestern, Central and Eastern Mediterranean Sea, respectively.

| main items and index | site | sub-basin | prey size | PPSR | selection of prey | niche width based on SCA | isotope niche (‰^2^) | source |
| --- | --- | --- | --- | --- | --- | --- | --- | --- |
| *Engraulis encrasicolus* |  |  |  |  |  |  |  |  |
| *Centropages typicus*, *Microsetella rosea, Oncaea* spp., *Temora stylifera* | Catalan Sea | NW Mediterranean Sea | from 0.54 mm (*Oncaea* spp.) to 1.24 mm (*C. typicus*) and 1.28mm (*T. stylifera*) |  |  |  |  | ^1^ |
| Copepods (main species consumed were *Candacia longimana*, *Oncaea mediterranea* and *Pleuromamma abdominalis*) constituted the dominant group (64% O; 81% N), other crustaceans, mollusc larvae and fish larvae were less represented. Other important species locally consumed were *Centropages chierchiae*, *Microsetella rosea*, and *Temora stylifera* | 3 study areas on the Algerian coast | SW Mediterranean | prey size varied from 0.57 mm (*Euterpina acutifrons*) to 6.8 mm (fish larvae) |  |  |  |  | ^2^ |
| *Euterpina acutifrons* (Harpacticoida) and *Oncaea* spp. (Poecilostomatoida) dominated the diet during the day, in terms of %O, %N and %W, in all the fish size classes. At night, bivalve larvae were important (IRI= 43.34, 40.22 and 24.86% for late larvae, juveniles and adults, respectively) and, with *Oncaea* spp. and *E. acutifrons*, made up >69% of total prey number for all anchovy size classes (%N = 76.52, 78.2 and 69.71% for late larvae, juveniles and adults, respectively). | off Pesaro (Marche Region) | N Adriatic Sea |  |  |  |  |  | ^3^ |
| Adults mostly fed on cladocerans (37.8%) and appendicularians (24.7%), juveniles fed primarily on cladocerans (33.8%) and copepods (35.5%). | Gulf of Lion | NW Mediterranean Sea |  |  | Ivlev’s selectivity index showed that during the summer, both cladocerans and appendicularians were the most heavily selected prey |  |  | ^4^ |
| Crustacean larvae (53.3%; dominant larval groups: Brachyura and Euphasiacea) were the most important taxon | N Aegean Sea | N Aegean Sea |  |  |  |  |  | ^5^ |
| *Acartia clausi*, *Oncaea* spp., *Euterpina acutifrons* and *Temora stylifera* were the main prey in summer. *Centropages* spp. were the dominant food item in winter, followed by Pontellidae and Clauso-Paracalanidae | N Aegean Sea | Aegean Sea | lengths of prey were mainly 401 to 800 μm and 1201 to 1400 μm |  | Positive selection for *Euterpina acutifrons* in summer and *Oncaea* spp. in both summer and winter. Negative selection for selec tivity values were calculated for taxa such as *Penilia avirostris* in summer or the Clauso-Paracalanidae group in both summer and winter. Rare taxa in the environment such as *Microsetella rosea* and *Candacia* spp. were positively selected |  |  | ^6^ |
| %IRI: *Microsetella* sp. (32.06), *Oncaea* sp. (25.25) and Corycaeidae (16.62). | Gulf of Lion | NW Mediterranean Sea | mainly 0.2-0.6 mm prey (and more 0.4-0.5) | 236.67 ± 133.66 |  | BG = 15.52 | mean SEA_B_ = 0.60 | ^7^ |
|  | off Ebro delta | NW Mediterranean Sea |  |  |  |  | SEA_B_=0-6-0.8 | ^8^ |
| %IRI), *Microsetella* spp. (13.52 %IRI), Clauso/Paracalanidae (13.22 %IRI), *Oncaea* spp. (12.67 %IRI) and Euphausiacaea larvae (7.31 %IRI). Copepods also dominated the anchovy diet in 2011-2012, but anchovy fed almost only on two species: *Microsetella* spp. (34.78 %IRI) and *Oncaea* spp. (27.52 %IRI). |  |  |  |  |  |  |  |  |
| The diet (based on SIA) mainly based on species of group 1 (*Centropages typicus*, *Pleuromamma gracilis*, *Temora stylifera* and *Corycaeus* spp.) , together with *Nyctiphanes couchi* and deapod larvae | Tyrrhenian Sea and Strait of Sicily | W and C Mediterranean Sea |  |  |  |  |  | ^9^ |
| The diet was mainly based on copepods (mostly *Temora stylifera*, Oncaeid copepods and Corycaeidae) The contribution of copepods decreased in larger individuals, and were gradually substituted by large crustaceans-decapods, euphausiids, mysids and amphipods | E Adriatic Sea | E Adriatic Sea |  |  |  |  |  | ^10^ |
| The diet (based on DNA metabarcoding on stomach contents) was based mainly on Euphausiids as well as other Malacostraca (including mysids and unidentified larvae and/or naupli)(60% of their total prey ingestion) in numbers. Their diet was completed mostly with copepods and with much less frequent other prey. | W Mediterranean Sea | W Mediterranean Sea |  |  | Anchovy completed their diet ingesting relatively smaller prey (i.e., copepods) in higher numbers. |  | SEA_C=_ 0.21 | ^11,12^ |
| Frequency of occurence (%F) and numerical abundance (%N): it ingested mainly copepods, Bivalvia larvae and fish ova/larvae | E Adriatic Sea | E Adriatic Sea |  |  |  | Levins’ standardized index ranged from 0.08 to 0.37 | SEA_C_= 0.54 | ^13^ |
| *Sardina pilchardus* |  |  |  |  |  |  |  |  |
| Adults had a heterogeneous diet, with appendicularians (29.2%) as the main prey. The diet of juveniles was based on cladocerans (36.8%), copepods (33.5%) and microplankton (25.1%) | Gulf of Lion | NW Mediterranean Sea |  |  | Ivlev’s selectivity index showed that during the summer, both cladocerans and appendicularians were the most heavily selected prey |  |  | ^4^ |
| Juveniles mainly fed on large calanoid copepods (*Temora stylifera* and *Acartia clausi*, *Euterpina acutifrons* and *Oncaea* spp.). Adults’ diet was numerically dominated by diatoms and dinoflagellates | N Aegean Sea | Aegean Sea |  |  | high selectivity (E) for specific taxa with low abundance in the field (e.g., *Microsetella rosea*, cirriped larvae, etc) |  |  | ^6^ |
| %IRI: cladocerans (51) and diatoms (47) in juveniles in summer and winter respectively; cladocerans (14), Oncoea (11), *Microsetella* spp. (9) in adults in summer, and diatoms (52) and other crustaceans (23) in winter respectively. | Gulf of Lion | NW Mediterranean Sea |  |  | Ivlev’s index: preference of juvenile sardines in summer for copepods (Harpacticoidae), and cladocerans, while in winter for *Candacia* spp., Corycaeidae and phytoplankton (radiolarians and diatoms); preference of adult sardines in summer for Corycaeidae, *Microsetella* spp. and cladocerans, while in winter for Corycaeidae, *Temora* spp., *Centropages* spp., cladocerans and appendicularians |  |  | ^14^ |
| Copepoda (42.9%) and Brachyuran larvae (39.9%) had the highest % weight contribution to the overall diet | N Aegean Sea | N Aegean Sea |  |  |  |  |  | ^5^ |
| %IRI: *Microsetella spp.* (28.63), *Oncaea* sp. (23.70) and Corycaeidae (20.25) | Gulf of Lion | NW Mediterranean Sea | mainly 0.2-0.6 mm prey (and more 0.4-0.5) | 259.47 ± 191.69 |  | BG = 16.67 | mean SEA_B_ = 0.75 | ^7^ |
|  | off Ebro delta | NW Mediterranean Sea |  |  |  |  | SEA_B_=0.8-1.4 | ^8^ |
| In 2007, diet was composed of copepods (60.31 %IRI, mainly *Euterpina acutifrons*, *Microsetella* spp. and *Temora stylifera*) and cladocerans (31.48 %IRI). Cladocerans were almost absent in 2011-2012, while *Microsetella* spp. (29.34 %IRI), *Oncaea* spp. (24.55 %IRI) and *Corycaeus* spp. (21.64 %IRI) dominated the sardine diet | Gulf of Lions | NW Mediterranean Sea |  |  |  |  | SEA_C_ ranged from 0.01 to 0.048 (from 2004 to 2014) | ^15^ |
| The diet (based on SIA) was mainly based on species of group 1 (*Centropages typicus*, *Pleuromamma gracilis*, *Temora stylifera* and *Corycaeus* spp.), together with *Paracalanus parvus* and *Acartia* sp. | Tyrrhenian Sea and Strait of Sicily | W and C Mediterranean Sea |  |  |  |  |  | ^9^ |
| Copepods (average contribution 30%), decapod larvae (18%), mysids (15%) and copepod eggs (9%) were the main food source. As fish size increased, copepods were gradually replaced in the diet by decapod larvae. In Spring, mysids were also important prey item of all size classes analysed, together with amphipods in the larger samples. Euphausiids were mostly consumed in summer, while autumn diet was mainly composed of copepods, copepods eggs and decapod larvae, especially in larger individuals. | E Adriatic Sea | Adriatic Sea |  |  |  |  |  | ^10^ |
| The diet (based on DNA metabarcoding on stomach contents) was predominately composed by copepods, comprising more than 50% of the prey abundance, followed by krill (32%). | Spanish waters | W Mediterranean Sea |  |  | In relation to fish size, the largest prey (i.e., euphausiids and decapods) were especially important in numbers in the diet of sardine |  | SEA_C=_ 0.38 | ^11,12^ |
| Frequency of occurence (%F) and numerical abundance (%N): it ingested mainly copepods, Bivalvia larvae and fish ova/larvae | E Adriatic Sea | Adriatic Sea |  |  |  | Levins’ standardized index ranged from 0.05 to 2 | SEA_C=_ 2.1 | ^13^ |
| %N: copepods (85.0) and Dinophyceae (9.5) in May; tintinnids (56.4), eggs and larvae of teleosts (12.2), Dinophyceae (11.3) and copepods (10.8) in June; copepods (48.4) and Crustacea larvae (21.1) in July; copepods (50.4) and chaetognaths (44.3) in October. | Gulf of Trieste | N Adriatic Sea |  |  | Ivlev index: preference of adult sardines for large prey (chaetognaths, decapod larvae, eggs and larvae of teleosts), but also some smaller prey such as harpacticoid and cyclopoid (*Ergasilida sp.*) copepods (with sizes of 150–880 micron and 125–625 micron, respectively) and cirriped larvae (220–660 micron) positively selected. |  |  | ^16^ |
| *Sprattus sprattus* |  |  |  |  |  |  |  |  |
| 69% of the total food was represented by *Temora stylifera*, *T. longicornis*, *Centropages typicus* and the genus *Calanus* | off Istria and Kvorner Bay | NE Adriatic Sea |  |  |  |  |  | ^17^ |
| %IRI: Clauso/Paracalanus (23.41), Corycaeidae (19.04), *Euterpina acutifrons* (14.61), *Microsetella spp.* (10.08) | Gulf of Lion | NW Mediterranean Sea | mainly 0.3-0.7 mm prey (and more 0.5-0.6) | 176.45 ± 71.14 |  | BG = 17.87 | mean SEA_B_ = 0.79 | ^7^ |
| *Acartia clausi*, *Euchaeta* sp., *Microcalanus* sp., *Calanus* spp., and cladocerans | W Adriatic Sea | W Adriatic Sea |  |  |  |  | mean SEAc=1.22 | this study |

**Table S2.** Results of the analyses conducted to determine the diet of *Sprattus sprattus*. a) overall food items ingested by *Sprattus sprattus* throughout the sampling period in the Northern and Southern Adriatic Sea (NA and SA, respectively) in terms of percentage by weight; b) results of the SIMPER analysis for similarity of samples within each size class and dissimilarity of samples among the two size classes for *Sprattus sprattus*. Cut-off for low contribution at 80%. Av. Abund = average abundance; Av. Sim = average similarity; Contrib %= percentage of variance explained by the explanatory variables; Cum. %= cumulative percentage of variance explained by the explanatory variables.

**a)**

| Taxon | NA | SA |
| --- | --- | --- |
| **CNIDARIA SIPHONOPHORA** |  |  |
| Unidentified siphonophores | 2.45 | - |
| **ANELLIDA POLYCHAETHA** |  |  |
| Unidentified polychaetes | 0.32 | 0.03 |
| **MOLLUSCA** |  |  |
| Unidentified bivalves | 1.95 | 4.21 |
| Unidentified gastropods | 0.06 | - |
| **ARTHROPODA CRUSTACEA** |  |  |
| **Copepoda** |  |  |
| *Calanus* sp.e | 13.84 | 10.37 |
| *Microcalanus* sp. | 29.10 | 23.76 |
| *Paracalanus* sp. | 0.64 | 18.20 |
| *Acartia clausi* | 9.94 | 5.33 |
| *Oncaea* sp. | 0.96 | 0.56 |
| *Euchaeta* sp. | 4.46 | 1.07 |
| *Centropages* sp. | 2.06 | 0.72 |
| *Aetideus* sp. | 0.35 | 0.50 |
| *Candacia* sp. | 1.15 | 0.81 |
| *Mecynocera* sp. | - | 0.09 |
| *Eurytemora* sp. | 0.20 | - |
| *Metridia* sp. | 0.08 | - |
| *Microsetella* sp. | - | 0.35 |
| Copepods remains | 13.97 | 13.54 |
| **Cladocera** |  |  |
| Unidentified cladocerans | 9.51 | 6.70 |
| **Amphipoda** |  |  |
| Unidentified hyperiids | 0.15 | - |
| **Isopoda** |  |  |
| Unidentified isopods | 0.95 | 0.04 |
| **Ostracoda** |  |  |
| Unidentified ostracods | 1.70 | 5.01 |
| **Decapoda** |  |  |
| Unidentified decapods | 0.90 | 1.00 |
| **OTHERS** |  |  |
| Eggs | 5.06 | 7.62 |
| Foraminifera | 0.20 | 0.09 |

**b)**

| Medium | Average similarity: 41,13 | | | | |
| --- | --- | --- | --- | --- | --- |
| Taxon | Av.Ab. | Av.Sim | Contrib% | Cum.% |  |
| *Microcalanus* sp. | 0.22 | 12.39 | 30.12 | 30.12 |  |
| *Calanus* sp. | 0.18 | 10.59 | 25.75 | 55.87 |  |
| Cladocerans | 0.12 | 4.43 | 10.77 | 66.64 |  |
| *Acartia* sp. | 0.10 | 3.90 | 9.49 | 76.14 |  |
| Large | Average similarity: 41,04 | | | | |
| *Microcalanus* sp. | 0.24 | 10.57 | 25.74 | 25.74 |  |
| *Acartia* sp. | 0.20 | 8.95 | 21.80 | 47.55 |  |
| *Calanus* sp. | 0.20 | 8.36 | 20.38 | 67.92 |  |
| Cladocerans | 0.16 | 4.96 | 12.08 | 80.00 |  |
| Medium vs. Large | Average dissimilarity = 60,65 | | | | |
| Taxon | Av.Ab. M | Av.Ab. L | Av.Diss | Contrib% | Cum.% |
| *Microcalanus* sp. | 0.22 | 0.24 | 6.50 | 10.72 | 10.72 |
| *Acartia* sp. | 0.10 | 0.20 | 6.38 | 10.52 | 21.24 |
| Cladocerans | 0.12 | 0.16 | 5.89 | 9.71 | 30.95 |
| *Calanus* sp. | 0.18 | 0.20 | 5.79 | 9.55 | 40.50 |
| Copepodes remains | 0.09 | 0.09 | 5.49 | 9.04 | 49.55 |
| Eggs | 0.07 | 0.09 | 4.64 | 7.65 | 57.19 |
| *Euchaeta* sp. | 0.03 | 0.08 | 3.43 | 5.66 | 62.85 |
| Decapods | 0.06 | 0.10 | 3.41 | 5.63 | 68.48 |
| Ostracods | 0.08 | 0.03 | 3.40 | 5.60 | 74.08 |

**Table S3.** Correlation between total length (TL) of the three small pelagic species (Sprspr=*Sprattus sprattus*, Engenc= *Engraulis encrasicolus* and Sarpil= *Sardina pilchardus*) and stable isotope ratios of nitrogen and carbon per each sub-area under study (NA=North Adriatic, CA=Central Adriatic, SA=South Adriatic). R=Pearson correlation coefficient.

|  | **Sprspr** | |  | **Engenc** | |  | **Sarpil** | |
| --- | --- | --- | --- | --- | --- | --- | --- | --- |
|  | ***δ*^15^N** | ***δ*^13^C** |  | ***δ*^15^N** | ***δ*^13^C** |  | ***δ*^15^N** | ***δ*^13^C** |
| **sub-area** | **NA** | |  | **NA** | |  | **NA** | |
| **R** | -0.07 | -0.12 |  | -0.39 | 0.61 |  | 0.20 | 0.43 |
| **p** | 0.56 | 0.33 |  | 0.0006 | < 0.0001 |  | 0.07 | 0.00006 |
|  | **CA** | |  | **CA** | |  | **CA** | |
| **R** |  |  |  | -0.69 | 0.31 |  | -0.77 | 0.26 |
| **p** |  |  |  | < 0.0001 | 0.006 |  | < 0.0001 | 0.14 |
|  | **SA** | |  | **SA** | |  | **SA** | |
| **R** | -0.02 | 0.15 |  | -0.65 | 0.34 |  | -0.48 | 0.27 |
| **p** | 0.96 | 0.67 |  | < 0.0001 | 0.03 |  | 0.03 | 0.24 |

**Table S4.** Results of multivariate (on *δ*^13^C-*δ*^15^N) and univariate (on *δ*^13^C and *δ*^15^N, separately) PERMANOVA main test (Design 1) (a). pairwise comparisons (b) for the interaction term ‘species × size’ for pairs of levels of factors ‘species’ of factor ‘size’ and (c) for the interaction term ‘species × size’ for pairs of levels of factors ‘size class’ of factor ‘species’. Sprspr = *Sprattus sprattus*; Sarpil= *Sardina pilchardus*; Engenc = *Engraulis encrasicolus.* df=degrees of freedom; MS= mean square; Pseudo-F= statistic F; t=statistic t for pairwise comparisons; Unique perms= number of permutations; P= probability level; n.s. = not significant.

| a) | *δ*^13^C-*δ*^15^N | | | |  | *δ*^15^N | | |  | *δ*^13^C | | |
| --- | --- | --- | --- | --- | --- | --- | --- | --- | --- | --- | --- | --- |
| Source | df | MS | Pseudo-F | P(perm) |  | MS | Pseudo-F | P(perm) |  | MS | Pseudo-F | P(perm) |
| Species | 2 | 42.23 | 29.96 | 0.0001 |  | 14.52 | 13.32 | 0.0001 |  | 27.71 | 86.63 | 0.0001 |
| Size | 1 | 7.74 | 5.49 | 0.0099 |  | 4.74 | 4.35 | 0.04 |  | 3.00 | 9.38 | 0.0024 |
| SpeciesxSize | 2 | 19.14 | 13.58 | 0.0001 |  | 11.35 | 10.42 | 0.0001 |  | 7.79 | 24.35 | 0.0001 |
| Residuals | 400 | 1.41 |  |  |  | 1.09 |  |  |  | 0.32 |  |  |
| Total | 405 |  |  |  |  |  |  |  |  |  |  |  |
| b) Term 'Speciesxsize' for pairs of levels of factor 'Species' | | | | | | | | | | | | |
| Within level 'M' of factor 'size class' | | | | | | | | | | | | |
| Groups |  |  | t | P(perm) |  |  | t | P(perm) |  |  | t | P(perm) |
| Sprspr, Sarpil |  |  | 5.48 | 0.0001 |  |  | 5.93 | 0.0001 |  |  | 4.90 | 0.0001 |
| Sprspr, Engenc |  |  | 2.13 | 0.0141 |  |  | 1.04 | n.s. |  |  | 2.78 | 0.0061 |
| Sarpil, Engenc |  |  | 6.35 | 0.0001 |  |  | 5.86 | 0.0001 |  |  | 7.01 | 0.0001 |
| Within level 'L' of factor 'size class' | | | | | | | | | | | | |
| Sprspr, Sarpil |  |  | 3.35 | 0.0005 |  |  | 3.34 | 0.0015 |  |  | 3.36 | 0.0011 |
| Sprspr, Engenc |  |  | 4.87 | 0.0001 |  |  | 2.60 | 0.0097 |  |  | 13.59 | 0.0001 |
| Sarpil, Engenc |  |  | 4.70 | 0.0001 |  |  | 0.52 | n.s. |  |  | 11.47 | 0.0001 |
| c) Term 'Spxsi' for pairs of levels of factor 'size class' | | | | | | | | | | | | |
| Within level 'Sprspr' of factor 'Species' | | | | | | | | | | | | |
| M vs L |  |  | 2.57 | 0.0039 |  |  | 1.31 | n.s. |  |  | 3.40 | 0.0012 |
| Within level 'Sarpil' of factor 'Species' | | | | | | | | | | | | |
| M vs L |  |  | 3.31 | 0.0004 |  |  | 1.62 | n.s. |  |  | 5.33 | 0.0001 |
| Within level 'Engenc' of factor 'Species' | | | | | | | | | | | | |
| M vs L |  |  | 4.51 | 0.0001 |  |  | 4.64 | 0.0001 |  |  | 3.54 | 0.0009 |

**Table S5.** Results of multivariate (on *δ*^13^C-*δ*^15^N) and univariate (on *δ*^13^C and *δ*^15^N, separately) PERMANOVA main test (Design 2) (a). pairwise comparisons and (b) for the interaction term ‘species × sub-basin’ for pairs of levels of factors ‘species’ of factor ‘species’. Sprspr = *Sprattus sprattus*; Sarpil= *Sardina pilchardus*; Engenc = *Engraulis encrasicolus*; NA= North Adriatic Sea; CA= Central Adriatic Sea; SA= South Adriatic Sea. df=degrees of freedom; MS= mean square; Pseudo-F= statistic F; t=statistic t for pairwise comparisons; Unique perms= number of permutations; P= probability level; n.s. = not significant.

| a) | *δ*^13^C-*δ*^15^N | | | |  | *δ*^15^N | | |  | *δ*^13^C | | |  |
| --- | --- | --- | --- | --- | --- | --- | --- | --- | --- | --- | --- | --- | --- |
| Source | df | MS | Pseudo-F | P(perm) |  | MS | Pseudo-F | P(perm) |  | MS | Pseudo-F | P(perm) |  |
| Species | 2 | 31.85 | 24.21 | 0.0001 |  | 15.59 | 15.76 | 0.0001 |  | 16.25 | 49.87 | 0.0001 |  |
| Sub-area | 2 | 16.50 | 12.54 | 0.0001 |  | 8.07 | 8.15 | 0.0003 |  | 8.43 | 25.85 | 0.0001 |  |
| SpeciesxSub-area | 3 | 11.33 | 8.62 | 0.0001 |  | 9.63 | 9.73 | 0.0001 |  | 1.70 | 5.23 | 0.0019 |  |
| Residuals | 398 | 1.32 |  |  |  | 0.99 |  |  |  | 0.33 |  |  |  |
| Total | 405 |  |  |  |  |  |  |  |  |  |  |  |  |
| b) Term 'Spxsu' for pairs of levels of factor 'Species' | | | | | | | | | | | | |  |
| Within level 'NA' of factor 'sub-area' | | | | | | | | | | | | |  |
| Groups |  |  | t | P(perm) |  |  | t | P(perm) |  |  | t | P(perm) |  |
| Sprspr, Sarpil |  |  | 3.55 | 0.0001 |  |  | 4.59 | 0.0001 |  |  | 0.12 | n.s. |  |
| Sprspr, Engenc |  |  | 4.32 | 0.0001 |  |  | 0.67 | n.s. |  |  | 7.96 | 0.0001 |  |
| Sarpil, Engenc |  |  | 5.24 | 0.0001 |  |  | 4.00 | 0.0002 |  |  | 7.38 | 0.0001 |  |
| Within level 'CA' of factor 'sub-area' | | | | | | | | | | | | |  |
| Sarpil, Engenc |  |  | 2.67 | 0.0051 |  |  | 0.92843 | n.s. |  |  | 7.1279 | 0.0001 |  |
| Within level 'SA' of factor 'sub-area' | | | | | | | | | | | | |  |
| Sprspr, Sarpil |  |  | 4.69 | 0.0001 |  |  | 5.57 | 0.0001 |  |  | 2.96 | 0.0047 |  |
| Sprspr, Engenc |  |  | 5.43 | 0.0001 |  |  | 5.93 | 0.0001 |  |  | 0.49 | n.s. |  |
| Sarpil, Engenc |  |  | 2.54 | 0.0056 |  |  | 0.93 | n.s. |  |  | 4.89 | 0.0001 |  |
| c) Term 'SpeciesxSub-area' for pairs of levels of factor 'sub-area'  Within level 'Sprspr' of factor 'Species' | | | | | | | | | | | | |  |
|  |  |  |  |  |  |  |  |  |  |  |  |  |  |
| Groups |  |  | t | P(perm) |  |  | t | P(perm) |  |  | t | P(perm) |  |
| NA, SA |  |  | 4.44 | 0.0001 |  |  | 2.09 | 0.0377 |  |  | 6.2955 | 0.0001 |  |
| Within level 'Sarpil' of factor 'Species' | | | | | | | | | | | | |  |
| NA, SA |  |  | 2.83 | 0.0009 |  |  | 3.13 | 0.0024 |  |  | 2.28 | 0.0252 |  |
| NA, CA |  |  | 1.84 | 0.0378 |  |  | 1.56 | n.s. |  |  | 2.26 | 0.0242 |  |
| SA, CA |  |  | 1.22 | n.s. |  |  | 1.48 | n.s. |  |  | 0.41 | n.s. |  |
| Within level 'Engenc' of factor 'Species' | | | | | | | | | | | | |  |
| NA, SA |  |  | 6.69 | 0.0001 |  |  | 7.43 | 0.0001 |  |  | 2.31 | 0.0234 |  |
| NA, CA |  |  | 3.45 | 0.0005 |  |  | 3.63 | 0.0005 |  |  | 2.13 | 0.0341 |  |
| SA, CA |  |  | 3.65 | 0.0002 |  |  | 3.80 | 0.0001 |  |  | 1.08 | n.s. |  |

**Table S6.** Results of multivariate (on *δ*^13^C-*δ*^15^N) and univariate (on *δ*^13^C and *δ*^15^N, separately) PERMANOVA main test (Design 3) (a) and the pairwise comparisons for the interaction term ‘species × inshore vs. offshore’ for pairs of levels of factors ‘species’ (b) and for pairs of levels of factors ‘inshore vs. offshore’(c). Sarpil= *Sardina pilchardus*; Engenc = *Engraulis encrasicolus.* df=degrees of freedom; MS= mean square; Pseudo-F= statistic F; t=statistic t for pairwise comparisons; Unique perms= number of permutations; P= probability level; n.s. = not significant.

| a) | *δ*^13^C-*δ*^15^N | | | |  | *δ*^15^N | | |  | *δ*^13^C | | |
| --- | --- | --- | --- | --- | --- | --- | --- | --- | --- | --- | --- | --- |
| Source | df | MS | Pseudo-F | P(perm) |  | MS | Pseudo-F | P(perm) |  | MS | Pseudo-F | P(perm) |
| Species | 1 | 30.66 | 22.81 | 0.00 |  | 2.25 | 2.22 | 0.13 |  | 28.40 | 86.05 | 0.0001 |
| In vs. off | 1 | 83.69 | 62.26 | 0.00 |  | 76.66 | 75.59 | 0.00 |  | 7.04 | 21.32 | 0.0001 |
| SpeciesxIn vs. off | 1 | 6.87 | 5.11 | 0.01 |  | 6.86 | 6.76 | 0.01 |  | 0.01 | 0.04 | n.s. |
| Residuals | 321 | 1.34 |  |  |  | 1.01 |  |  |  | 0.33 |  |  |
| Total | 324 |  |  |  |  |  |  |  |  |  |  |  |
| b) Term 'SpeciesxIn vs. off' for pairs of levels of factor 'Species' | | | | | | | | | | | | |
| Within level 'in' of factor 'in vs. off' | | | | | | | | | | | | |
| Groups |  |  | t | P(perm) |  |  | t | P(perm) |  |  | t | P(perm) |
| Sarpil, Engenc |  |  | 6.11 | 0.0001 |  |  | 4.33 | 0.0001 |  |  | 9.26 | 0.0001 |
| Within level 'off' of factor 'in vs. off' | | |  |  |  |  |  |  |  |  |  |  |
| Sarpil, Engenc |  |  | 2.87 | 0.0016 |  |  | 0.65 | n.s. |  |  | 7.78 | 0.0001 |
| c) Term 'SpeciesxIn vs. off' for pairs of levels of factor 'in vs. off' | | | | | | | | | | | | |
| Within level 'Sarpil' of factor 'Species' | | | | | | | | | | | | |
| in, off |  |  | 3.62 | 0.0001 |  |  | 4.20 | 0.0002 |  |  | 2.42 | 0.0187 |
| Within level 'Engenc' of factor 'Species' | | | | | | | | | | | | |
| in, off |  |  | 8.77 | 0.0001 |  |  | 9.32 | 0.0001 |  |  | 4.79 | 0.0001 |

**Table S7.** Values of Total Area of the convex hull (TA) and of the corrected Standard Ellipse Area (SEA_C_) obtained considering the four different “ancillary” species and the areas in which they were caught. Values of TA and SEA_C_ are expressed as ‰^2^. Other values are shown in the table: *δ*^15^N range (NR), *δ*^13^C range (CR), and the mean distance to centroid (CD).

| **Species** | ***S. sprattus*** | | ***S. pilchardus*** | | | ***E. encrasicolus*** | | |
| --- | --- | --- | --- | --- | --- | --- | --- | --- |
| TA | 6.64 | | 10.17 | | | 10.29 | | |
| SEA_C_ | 1.22 | | 2.14 | | | 2.01 | | |
| NR | 0.43 | | 0.66 | | | 1.48 | | |
| CR | 1.21 | | 0.47 | | | 0.25 | | |
| CD | 0.60 | | 0.30 | | | 0.54 | | |
| **Area** | **NA** | **SA** | **NA** | **CA** | **SA** | **NA** | **CA** | **SA** |
| TA | 7.50 | 2.49 | 9.51 | 6.58 | 4.62 | 9.26 | 5.86 | 2.93 |
| SEA_C_ | 1.86 | 1.60 | 2.14 | 2.01 | 1.85 | 2.46 | 1.47 | 0.84 |

**Table S8.** Isotopic signatures of the sources used to construct the mixing polygon for each one of the three studied species in the different sub areas and to select the sources to be used to run SIMMR models. The complete stable isotope dataset on zooplankton cited in Fanelli et al., 2022 is available at [https://doi.org/10.1594/PANGAEA.945541](https://doi.pangaea.de/10.1594/PANGAEA.945541). s.d. = standard deviation.

| **Sub-area** | **Source** | **mean *δ*^13^C** | **s.d.** | **mean *δ*^15^N** | **s.d.** | **Isotopic signatures taken from** |
| --- | --- | --- | --- | --- | --- | --- |
| North Adriatic | *Acartia* sp. | -21.58 | 0.61 | 7.86 | 0.86 | ^18^ |
|  | *Centropages typicus* | -21.52 |  | 6.93 |  | ^18^ |
|  | *Oithona* sp. | -20.41 |  | 4.10 |  | ^18^ |
|  | Decapoda larvae | -19.81 | 0.19 | 7.58 | 1.45 | ^18^ |
|  | Large Copepoda | -16.39 | 0.41 | 7.19 | 0.12 | ^18^ |
|  | POM_NA | -23.05 | 0.20 | 10.00 | 0.20 | ^19^ |
|  | Phyto1 | -19.05 | 1.34 | 3.75 | 0.07 | ^20^ |
|  | Macroaggregates | -19.70 | 0.20 | 8.30 | 0.20 | ^20^ |
|  | Phyto2 | -23.90 | 0.71 | 3.60 | 0.85 | ^20^ |
| Central Adriatic | Clauso-Paracalanidae | -20.86 | 0.36 | 4.03 | 0.33 | ^18^ |
|  | *Calanus* spp. | -20.89 | 0.44 | 5.61 | 0.26 | ^18^ |
|  | *Centropages typicus* | -21.20 | 0.40 | 4.92 | 1.25 | ^18^ |
|  | Fish larvae | -20.57 | 0.26 | 5.09 | 0.53 | ^18^ |
|  | Decapoda larvae | -20.74 | 0.04 | 5.77 | 0.07 | ^18^ |
|  | *Temora* sp. | -20.56 |  | 4.71 |  | ^18^ |
|  |  | -19.17 | 0.07 | 3.89 | 0.06 | ^18^ |
|  | *Euchaeta* sp. | -21.03 | 0.26 | 5.42 | 0.50 | ^18^ |
|  | POM_NA | -23.05 | 0.20 | 10.00 | 0.20 | ^19^ |
|  | Phyto1 | -19.05 | 1.34 | 3.75 | 0.07 | ^20^ |
|  | Macroaggregates | -19.70 | 0.20 | 8.30 | 0.20 | ^20^ |
|  | Phyto2 | -23.90 | 0.71 | 3.60 | 0.85 | ^20^ |
| South Adriatic | Clauso-Paracalanidae | -20.64 | 0.03 | 3.98 | 0.42 | ^18^ |
|  | *Calanus* spp. | -21.04 | 0.92 | 6.54 | 0.66 | ^18^ |
|  | *Euchaeta* sp. | -20.93 | 0.29 | 5.08 | 0.20 | ^18^ |
|  | Decapoda larvae | -19.64 | 0.08 | 6.81 | 0.17 | ^18^ |
|  | *Temora stylifera* | -19.77 |  | 6.24 |  | ^18^ |
|  | *Pleuromamma abdominalis* | -21.14 |  | 3.59 |  | ^18^ |
|  | Thaliacea | -19.59 | 0.29 | 3.35 | 0.07 | ^18^ |
|  | Large Copepoda | -18.09 | 0.19 | 7.12 | 0.10 | ^18^ |
|  | POM_GT | -21.00 | 1.92 | 5.80 | 1.41 | ^20^ |
|  | POM_NA | -23.05 | 0.20 | 10.00 | 0.20 | ^19^ |
|  | Phyto1 | -19.05 | 1.34 | 3.75 | 0.07 | ^20^ |
|  | Macroaggregates | -19.70 | 0.20 | 8.30 | 0.20 | ^20^ |
|  | Phyto2 | -23.90 | 0.71 | 3.60 | 0.85 | ^20^ |

**Figure S1.** Cumulative prey curve for *Sprattus sprattus*, obtained on the number of taxa observed in the stomach contents and by bootstrapping.

**
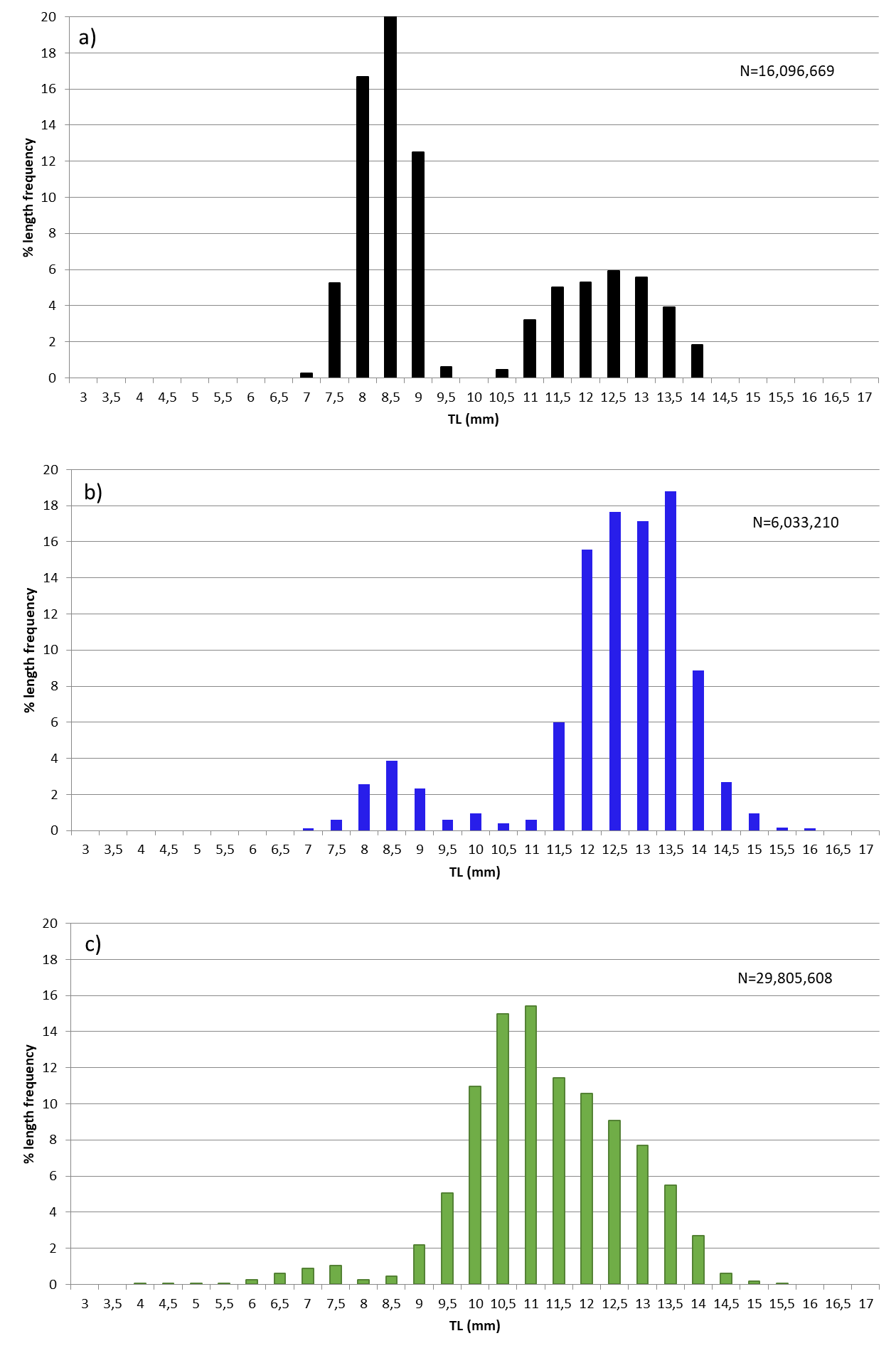
**

**Figure S2.** Length Frequency Distribution of a) *Sprattus sprattus*, b) *Sardina pilchardus* and c) *Engraulis encrasicolus* collected during MEDIAS 2019 survey in June-July 2019 in the Adriatic basin.


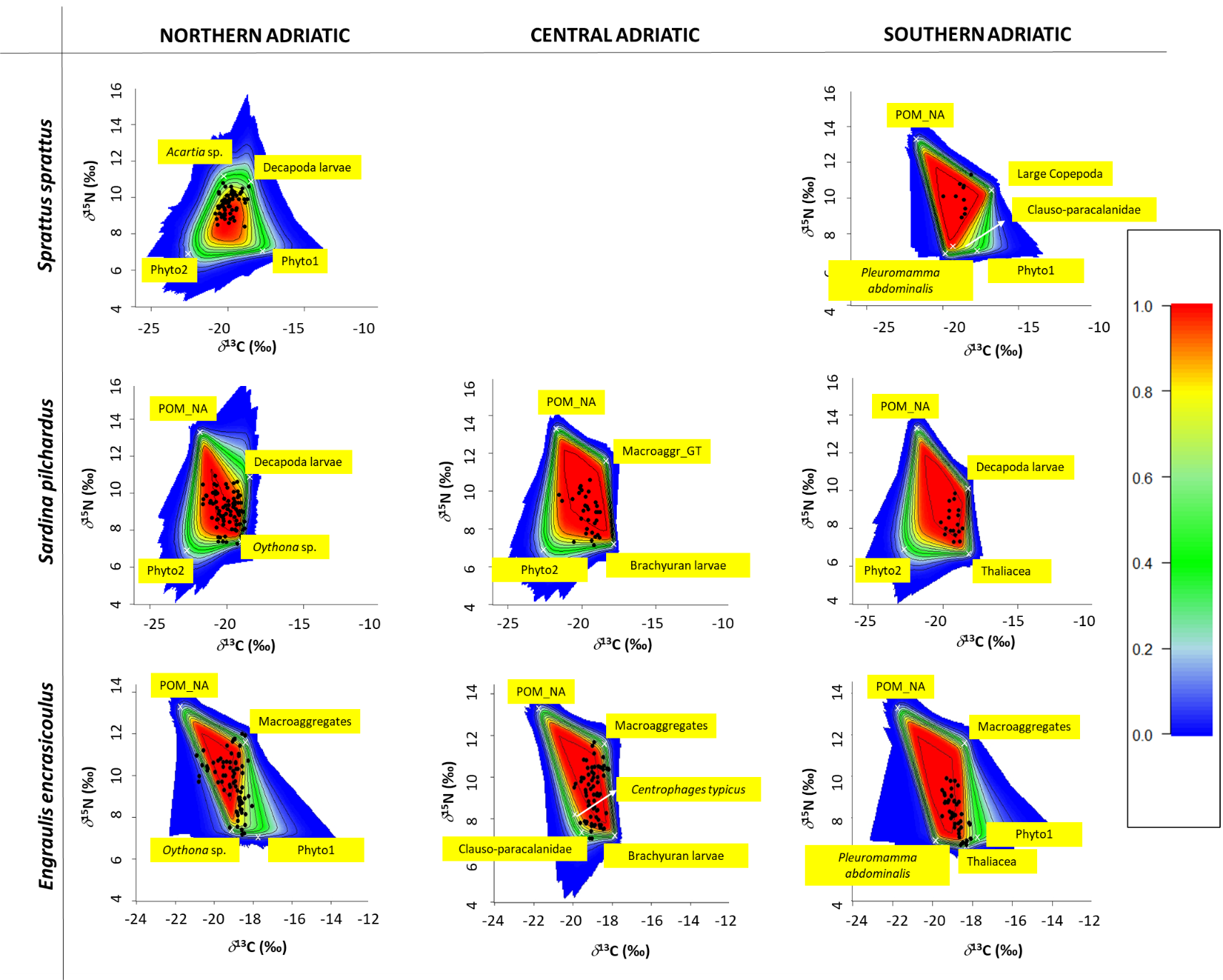


**Figure S3.** The biplots show the simulated mixing with the positions of the *Sprattus sprattus*, *Sardina pilchardus* and *Engraulis encrasicolus* samples (black dots) in the different sub-areas and the average signatures of the sources selected for the mixing model (white crosses). Probability contours are at the 5% level (outermost contour) and at every 10% level, following the colors shown in the legend in the right part of the graph. Macroaggregates = macroaggregates from the Northern Adriatic ^20^, POM_NA = POM (Particulate Organic Matter) from the Northern Adriatic ^19^, Phyto1 and Phyto2= phytoplankton from the Northern Adriatic ^20^.

References

1 Tudela, S. & Palomera, I. Trophic ecology of the European anchovy Engraulis encrasicolus in the Catalan Sea (northwest Mediterranean). *Marine Ecology Progress Series* **160**, 121-134 (1997).

2 Bacha, M. & Amara, R. Spatial, temporal and ontogenetic variation in diet of anchovy (*Engraulis encrasicolus*) on the Algerian coast (SW Mediterranean). *Estuarine, Coastal and Shelf Science* **85**, 257-264, https://doi.org/10.1016/j.ecss.2009.08.009 (2009).

3 Borme, D., Tirelli, V., Brandt, S. B., Fonda Umani, S. & Arneri, E. Diet of *Engraulis encrasicolus* in the northern Adriatic Sea (Mediterranean): ontogenetic changes and feeding selectivity. *Marine Ecology Progress Series* **392**, 193-209, https://doi.org/10.3354/meps08214 (2009).

4 Costalago, D., Navarro, J., Alvarez-Calleja, I. & Palomera, I. Ontogenetic and seasonal changes in the feeding habits and trophic levels of two small pelagic fish species. *Marine Ecology Progress Series*, 10.3354/meps09751 (2012).

5 Karachle, P. K. & Stergiou, K. I. An update on the feeding habits of fish in the Mediterranean Sea (2002-2015). *Mediterranean Marine Science* **18**, 43-52, 10.12681/mms.1968 (2017).

6 Nikolioudakis, N., Isari, S. & Somarakis, S. Trophodynamics of anchovy in a non-upwelling system: direct comparison with sardine. *Marine Ecology Progress Series* **500**, 215-229 (2014).

7 Le Bourg, B. *et al.* Trophic niche overlap of sprat and commercial small pelagic teleosts in the Gulf of Lions (NW Mediterranean Sea). *Journal of Sea Research* **103**, 138-146, https://doi.org/10.1016/j.seares.2015.06.011 (2015).

8 Albo-Puigserver, M., Navarro, J., Coll, M., Layman, C. A. & Palomera, I. Trophic structure of pelagic species in the northwestern Mediterranean Sea. *Journal of Sea Research* **117**, 27-35, https://doi.org/10.1016/j.seares.2016.09.003 (2016).

9 Rumolo, P. *et al.* Spatial variations in feeding habits and trophic levels of two small pelagic fish species in the central Mediterranean Sea. *Marine Environmental Research* **115**, 65-77, https://doi.org/10.1016/j.marenvres.2016.02.004 (2016).

10 Hure, M. & Mustać, B. Feeding ecology of *Sardina pilchardus* considering co-occurring small pelagic fish in the eastern Adriatic Sea. *Marine Biodiversity* **50**, https://doi.org/10.1007/s12526-020-01067-7 (2020).

11 Bachiller, E. *et al.* Trophic niche overlap between round sardinella (*Sardinella aurita*) and sympatric pelagic fish species in the Western Mediterranean. *Ecology and Evolution* **11**, 16126-16142, https://doi.org/10.1002/ece3.8293 (2021).

12 Bachiller, E. *et al.* A trophic latitudinal gradient revealed in anchovy and sardine from the Western Mediterranean Sea using a multi-proxy approach. *Sci Rep* **10**, 17598, 10.1038/s41598-020-74602-y (2020).

13 Zorica, B. *et al.* Diet Composition and Isotopic Analysis of Nine Important Fisheries Resources in the Eastern Adriatic Sea (Mediterranean). *Frontiers in Marine Science* **8**, https://doi.org/10.3389/fmars.2021.609432 (2021).

14 Costalago, D. & Palomera, I. Feeding of European pilchard (*Sardina pilchardus*) in the northwestern Mediterranean: from late larvae to adults. *Scientia Marina* **78**, 41-54, https://doi.org/10.3989/scimar.03898.06D (2014).

15 Brosset, P. *et al.* Linking small pelagic dietary shifts with ecosystem changes in the Gulf of Lions. *Marine Ecology Progress Series* **554**, 157-171, 10.3354/meps11796 (2016).

16 Borme, D., Legovini, S., de Olazabal, A. & Tirelli, V. Diet of Adult Sardine *Sardina pilchardus* in the Gulf of Trieste, Northern Adriatic Sea. *Journal of Marine Science and Engineering* **10**, 10.3390/jmse10081012 (2022).

17 Tičina, V., Vidjak, O. & Kačič, I. Feeding of adult sprat*, Sprattus sprattus* ,during spawning season in the Adriatic Sea. *Italian Journal of Zoology* **67**, 307-311, https://doi.org/10.1080/11250000009356329 (2000).

18 Fanelli, E., Menicucci, S., Malavolti, S., De Felice, A. & Leonori, I. Mesoscale variations in the assemblage structure and trophodynamics of mesozooplankton communities of the Adriatic basin (Mediterranean Sea). *Biogeosciences*, https://doi.org/10.5194/bg-2021-240 (2022).

19 Berto, D. *et al*. Stable carbon and nitrogen isotope ratios as tools to evaluate the nature of particulate organic matter in the Venice lagoon. *Estuarine, Coastal and Shelf Science* **135**, 66-76, https://doi.org/10.1016/j.ecss.2013.06.021 (2013).

20 Faganeli, J. *et al.* Carbon and nitrogen isotope composition of particulate organic matter in relation to mucilage formation in the northern Adriatic Sea. *Marine Chemistry* **114**, 102-109, https://doi.org/10.1016/j.marchem.2009.04.005 (2009).
